# A computational theory of visual receptive fields

**DOI:** 10.1007/s00422-013-0569-z

**Published:** 2013-11-07

**Authors:** Tony Lindeberg

**Affiliations:** Department of Computational Biology, School of Computer Science and Communication, KTH Royal Institute of Technology, 100 44 Stockholm, Sweden

**Keywords:** Receptive field, Scale space, Gaussian derivative, Scale covariance, Affine covariance, Galilean covariance, Illumination invariance, LGN, Primary visual cortex, Visual area V1, Functional model, Simple cell, Double-opponent cell, Complex cell, Vision, Theoretical neuroscience, Theoretical biology

## Abstract

A receptive field constitutes a region in the visual field where a visual cell or a visual operator responds to visual stimuli. This paper presents a theory for what types of receptive field profiles can be regarded as natural for an *idealized vision system*, given a set of *structural requirements* on the first stages of visual processing that reflect *symmetry properties* of the surrounding world. These symmetry properties include (i) *covariance properties* under scale changes, affine image deformations, and Galilean transformations of space–time as occur for real-world image data as well as specific requirements of (ii) *temporal causality* implying that the future cannot be accessed and (iii) a *time-recursive updating* mechanism of a limited temporal buffer of the past as is necessary for a genuine real-time system. Fundamental structural requirements are also imposed to ensure (iv) mutual consistency and a proper handling of internal representations at *different spatial and temporal scales*. It is shown how a set of *families of idealized receptive field profiles can be derived by necessity* regarding *spatial, spatio-chromatic, and spatio-temporal receptive fields* in terms of Gaussian kernels, Gaussian derivatives, or closely related operators. Such image filters have been successfully used as a *basis* for expressing a large number of visual operations in computer vision, regarding feature detection, feature classification, motion estimation, object recognition, spatio-temporal recognition, and shape estimation. Hence, the associated so-called *scale-space theory* constitutes a both theoretically well-founded and general framework for expressing visual operations. There are very close similarities between receptive field profiles predicted from this scale-space theory and receptive field profiles found by cell recordings in *biological vision*. Among the family of receptive field profiles derived by necessity from the assumptions, idealized models with very good qualitative agreement are obtained for (i) spatial on-center/off-surround and off-center/on-surround receptive fields in the fovea and the LGN, (ii) simple cells with spatial directional preference in V1, (iii) spatio-chromatic double-opponent neurons in V1, (iv) space–time separable spatio-temporal receptive fields in the LGN and V1, and (v) non-separable space–time tilted receptive fields in V1, all within the same unified theory. In addition, the paper presents a more general framework for relating and interpreting these receptive fields conceptually and possibly predicting new receptive field profiles as well as for pre-wiring covariance under scaling, affine, and Galilean transformations into the representations of visual stimuli. This paper describes the basic structure of the necessity results concerning receptive field profiles regarding the mathematical foundation of the theory and outlines how the proposed theory could be used in further studies and modelling of biological vision. It is also shown how receptive field responses can be *interpreted physically*, as the superposition of relative variations of surface structure and illumination variations, given a logarithmic brightness scale, and how receptive field measurements will be *invariant* under multiplicative illumination variations and exposure control mechanisms.

## Introduction

**Fig. 1:**
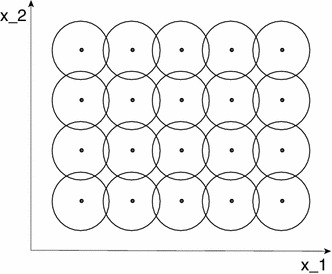
A receptive field is a region in the visual field for which a visual sensor/neuron/operator responds to visual stimuli. This *figure* shows a set of partially overlapping receptive fields over the spatial domain with all the receptive fields having the same spatial extent. More generally, one can conceive distributions of receptive fields over space or space–time with the receptive fields of different size, different shape, and orientation in space as well as different directions in space–time, where adjacent receptive fields may also have significantly larger relative overlap than shown in this schematic illustration

When light reaches a visual sensor such as the retina, the information necessary to infer properties about the surrounding world is not contained in the measurement of image intensity at a single point, but from the *relationships* between intensity values at different points. A main reason for this is that the incoming light constitutes an *indirect* source of information depending on the interaction between geometric and material properties of objects in the surrounding world and on external illumination sources. Another fundamental reason why cues to the surrounding world need to be collected over *regions* in the visual field as opposed to at single image points is that the measurement process by itself requires the accumulation of energy over non-infinitesimal support regions over space and time. Such a region in the visual field for which a visual sensor and or a visual operator responds to visual input or a visual cell responds to visual stimuli is naturally referred to as a *receptive field* (Hubel and Wiesel 1959, 1962) (see Fig. 1).

If one considers the theoretical and algorithmic problems of designing a vision system that is going to make use of incoming reflected light to infer properties of the surrounding world, one may ask what types of image operations should be performed on the image data. Would any type of image operation be reasonable? Specifically, regarding the notion of receptive fields, one may ask what types of receptive field profiles would be reasonable? Is it possible to derive a theoretical model of how receptive fields “ought to” respond to visual data?

Initially, such a problem might be regarded as intractable unless the question can be further specified. It is, however, possible to study this problem systematically using approaches that have been developed in the area of computer vision known as *scale-space theory* (Iijima 1962; Witkin 1983; Koenderink 1984; Koenderink and Doorn 1992; Lindeberg 1994a, b, 2008; Sporring et al. 1996; Florack 1997; ter Haar Romeny 2003). A paradigm that has been developed in this field is to impose *structural constraints* on the first stages of visual processing that reflect *symmetry properties* of the environment. Interestingly, it turns out to be possible to substantially reduce the class of permissible image operations from such arguments.

The subject of this article is to describe how structural requirements on the first stages of visual processing as formulated in scale-space theory can be used for deriving idealized models of receptive fields and implications of how these theoretical results can be used when modelling biological vision. A main theoretical argument is that idealized models for linear receptive fields can be derived *by necessity* given a small set of symmetry requirements that reflect properties of the world that one may naturally require an idealized vision system to be adapted to. In this respect, the treatment bears similarities to approaches in theoretical physics, where symmetry properties are often used as main arguments in the formulation of physical theories of the world. The treatment that will follow will be general in the sense that *spatial, spatio-chromatic, and spatio-temporal receptive fields are encompassed by the same unified theory*.

**Fig. 2:**
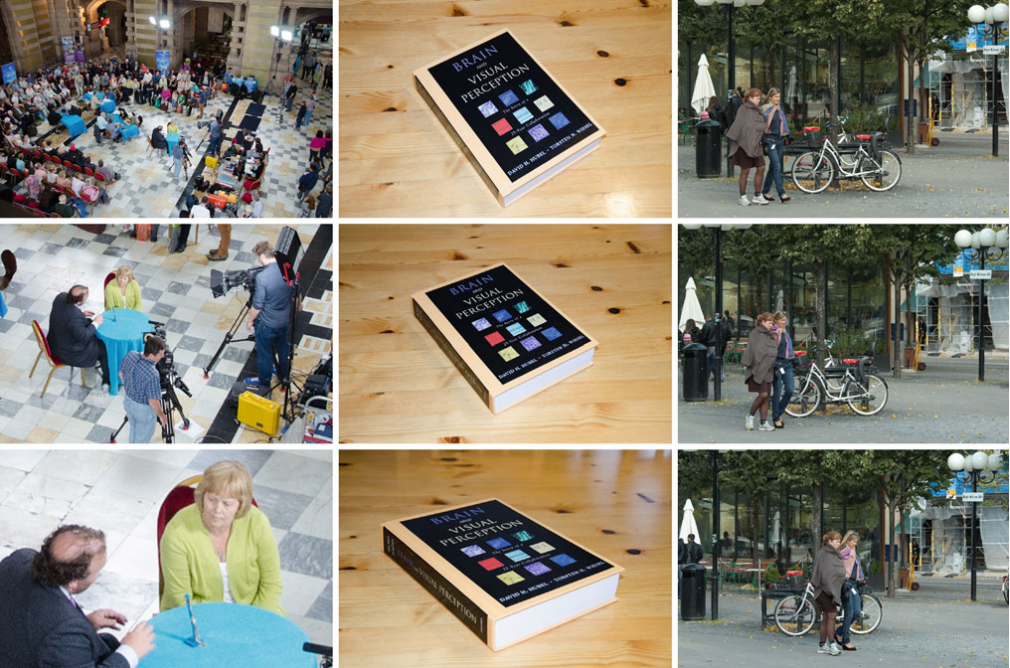
Visual stimuli may vary substantially on the retina due to geometric transformations and lighting variations in the environment. Nevertheless, the brain is able to perceive the world as stable. This figure illustrates examples of *natural image transformations* corresponding to (*left column*) variations in scale, (*middle column*) variations in viewing direction, and (*right column*) relative motion between objects in the world and the observer. A main subject of this paper is to present a theory for visual receptive fields that make it possible to match receptive field responses between image data that have been acquired under different image conditions, specifically involving these basic types of natural image transformations. To model the influence of natural image transformations on receptive field responses, we first approximate the possibly nonlinear image transformation by a local linear transformation at each image point (the derivative), which for these basic image transformations correspond to (i) local scaling transformations, (ii) local affine transformations, and (iii) local Galilean transformations. Then, we consider families of receptive fields that have the property that the transformation of any receptive field within the family using a locally linearized image transformation within the group of relevant image transformations is still within the same family of receptive fields. Such receptive field families are referred to as *covariant* receptive fields. The receptive field family is also said to be *closed* under the relevant group of image transformations

**Fig. 3:**
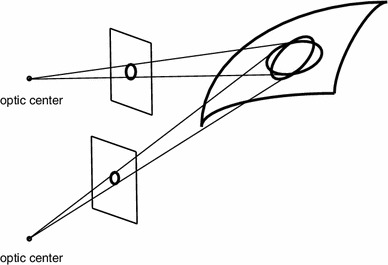
Consider a vision system that is restricted to using rotationally symmetric image operations over the spatial image domain only. If such a vision system observes the same three-dimensional object from two different views, then the backprojections of the receptive fields onto the surface of the object will in general correspond to different regions in physical space over which corresponding information will be weighed differently. If such image measurements would be used for deriving correspondences between the two views or performing object recognition, then there would be a systematic error caused by the mismatch between the backprojections of the receptive fields from the image domain onto the world. By requiring the family of receptive fields to be covariant under local affine image deformations, it is possible to reduce this amount of mismatch, such that the backprojected receptive fields can be made similar when projected onto the tangent plane of the surface by local linearizations of the perspective mapping. Corresponding effects occur when analyzing spatio-temporal image data (video) based on receptive fields that are restricted to being space–time separable only. If an object is observed over time by two cameras having different relative motions between the camera and the observer, then the corresponding receptive fields cannot be matched unless the family of receptive fields possesses sufficient covariance properties under local Galilean transformations

An underlying motivation for the theory is that due to the properties of the projection of three-dimensional objects to a two-dimensional light sensor (retina), the image data will be subject to basic image transformations in terms of (i) local *scaling transformations* caused by objects of different sizes and at different distances to the observer, (ii) local *affine transformations* caused by variations in the viewing direction relative to the object, (iii) local *Galilean transformations* caused by relative motions between the object and the observer, and (iv) local *multiplicative intensity transformations* caused by illumination variations (see Fig. 2). If the vision system is to maintain a stable perception of the environment, it is natural to require the first stages of visual processing to be robust to such image variations. Formally, one may require the receptive fields to be *covariant* under basic image transformations, which means that the receptive fields should be transformed in a well-behaved and well-understood manner under corresponding image transformations (see Fig. 3). Combined with an additional criterion that the receptive field must not create new structures at coarse scales that do not correspond to simplifications of corresponding finer scale structures, we will describe how these requirements together lead to idealized families of receptive fields (Lindeberg 2011) in good agreement with receptive field measurements reported in the literature (Hubel and Wiesel 1959, 1962; DeAngelis et al. 1995; DeAngelis and Anzai 2004; Conway and Livingstone 2006).

Specifically, explicit *functional models* will be given of spatial and spatio-temporal response properties of LGN neurons and simple cells in V1, which will be compared to related models in terms of Gabor functions (Marcelja 1980; Jones and Palmer 1987b, a), differences of Gaussians (Rodieck 1965), and Gaussian derivatives (Koenderink and Doorn 1987; Young 1987; Young et al. 2001; Young RA, Lesperance 2001; Lindeberg 1994a, b, 1997, 2011). For chromatic input, the model also accounts for color-opponent spatio-chromatic cells in V1. Notably, the diffusion equations that describe the evolution properties over scale of these linear receptive field models are suitable for implementation on a biological architecture, since the computations can be expressed in terms of communications between neighboring computational units, where either a single computational unit or a group of computational units may be interpreted as corresponding to a neuron or a group of neurons.

Compared to previous approaches of learning receptive field properties and visual models from the statistics of natural image data (Field 1987; van der Schaaf and van Hateren 1996; Olshausen and Field 1996; Rao and Ballard 1998; Simoncelli and Olshausen 2001; Geisler 2008; Hyvärinen et al. 2009; Lörincz et al. 2012), the proposed theoretical model makes it possible to determine spatial and spatio-temporal receptive fields from first principles and thus without need for any explicit training stage or gathering of representative image data. In relation to such learning-based models, the proposed theory provides a *normative* approach that can be seen as describing the solutions that an ideal learning-based system may converge to, if exposed to a sufficiently large and representative set of natural image data. For these reasons, the presented approach should be of interest when modelling biological vision.

We will also show how receptive field responses can be *interpreted physically* as a superposition of relative variations of surface structure and illumination variations, given a logarithmic brightness scale, and how receptive field measurements will be *invariant* under multiplicative illumination variations and exposure control mechanisms. Despite the image measurements fundamentally being of an *indirect* nature, in terms of reflected light from external objects subject to unknown or uncontrolled illumination, this result shows how receptive field measurements can nevertheless be related to *inherent physical properties of objects* in the environment. This result therefore provides a formal justification for using receptive field responses as a basis for visual processes, analogous to the way linear receptive fields in the fovea, LGN and V1 provide the basic input to higher visual areas in biological vision.

We propose that these theoretical results contribute to an increased understanding of the role of early receptive fields in vision. Specifically, if one aims at building a neuro-inspired artificial vision system that solves actual visual tasks, we argue that an approach based on the proposed idealized models of linear receptive fields should require a significantly lower amount of training data compared to approaches that involve specific learning of receptive fields or compared to approaches that are not based on covariant receptive field models. We also argue that the proposed families of covariant receptive fields will be better at handling natural image transformations as resulting from variabilities in relation to the surrounding world.

In their survey of our knowledge of the early visual system, Carandini et al. (2005) emphasize the need for functional models to establish a link between neural biology and perception. Einhäuser and König (2010) argue for the need for normative approaches in vision. This paper can be seen as developing the consequences of such ways of reasoning by deriving *functional models of linear receptive fields* using a normative approach. Due to the formulation of the resulting receptive fields in terms of spatial and spatio-temporal derivatives of convolution kernels, it furthermore becomes feasible to analyze how receptive field responses can be related to properties of the environment using mathematical tools from differential geometry and thereby analyzing possibilities as well as constraints for visual perception.

### Outline of the presentation

The treatment will be organized as follows: Sect. 2 formulates a set of structural requirements on the first stages of visual processing with respect to symmetry properties of the surrounding world and in relation to internal representations that are to be computed by an *idealized vision system*. Then, Sect. 3 describes the consequences of these assumptions with regard to intensity images defined over a spatial domain, with extensions to color information in Sect. 4. Sect. 5 develops a corresponding theory for spatio-temporal image data, taking into account the special nature of time-dependent image information.

Section 6 presents a comparison between spatial and spatio-temporal receptive fields measured from biological vision to receptive field profiles generated by the presented spatial, spatio-chromatic, and spatio-temporal scale-space theories, showing a very good qualitative agreement. Section 7 describes how a corresponding foveal scale-space model can be formulated for a foveal sensor to account for a spatially dependent lowest resolution with suggestions for extensions in Sect. 8.

Section 9 relates the contributions in the paper to previous work in the area in a retrospective manner, and Sect. 10 concludes with a summary and discussion, including an outline of further applications of how the presented theory can be used for modelling biological vision.

## Structural requirements of an idealized visual front end

The notion of a *visual front end* refers to a set of processes at the first stages of visual processing, which are assumed to be of a general nature and whose output can be used as input to different later-stage processes, without being too specifically adapted to a particular task that would limit the applicability to other tasks. Major arguments for the definition of a visual front end are that the first stages of visual processing should be as *uncommitted* as possible and allow initial processing steps to be *shared* between different later-stage visual modules, thus implying a *uniform structure* on the first stages of visual computations (Koenderink et al. 1992; Lindeberg 1994b, Sect. 1.1).

In the following, we will describe a set of structural requirements that can be stated concerning (i) spatial geometry, (ii) spatio-temporal geometry, (iii) the image measurement process with its close relationship to the notion of scale, (iv) internal representations of image data that are to be computed by a general purpose vision system, and (v) the parameterization of image intensity with regard to the influence of illumination variations.

The treatment that will follow can be seen as a unification, abstraction and extension of developments in the area of *scale-space theory* (Iijima 1962; Witkin 1983; Koenderink 1984; Koenderink and Doorn 1992; Lindeberg 1994a, b, 2008; Sporring et al. 1996; Florack 1997; ter Haar Romeny 2003) as obtained during the last decades, see Sect. 9.2 and (Lindeberg 1996, 2011; Weickert et al. 1999; Duits et al. 2004) for complementary surveys. It will then be shown how a generalization of this theory to be presented next can be used for deriving idealized models of receptive fields by necessity, including new extensions for modelling illumination variations in the intensity domain. Specifically, we will describe how these results can be used for computational neuroscience modelling of receptive fields with regard to biological vision.

### Static image data over spatial domain

Let us initially restrict ourselves to static (time-independent) data and focus on the spatial aspects: If we regard the incoming image intensity $f$ as defined on a 2D image plane $f :{\mathbb R}^2 \rightarrow {\mathbb R}$ with Cartesian image coordinates^1^ denoted by $x = (x_1, x_2)^T$, then the problem of defining a set of early visual operations can be formulated in terms of finding a family of operators $\mathcal{T}_s$ that are to act on $f$ to produce a family of new intermediate image representations^2^

1 $$\begin{aligned} L(\cdot ;\; s) = \mathcal{T}_s f \end{aligned}$$

which are also defined as functions on ${\mathbb R}^2$, i.e., $L(\cdot ;\; s) :{\mathbb R}^2 \!\rightarrow \! {\mathbb R}$. These intermediate representations may be dependent on some parameter $s$, which in the simplest case may be one-dimensional or under more general circumstances multi-dimensional.

#### Linearity and convolution structure

If we want these the initial visual processing stages to make as few irreversible decisions as possible, it is natural to initially require $\mathcal{T}_s$ to be a *linear operator* such that^3^

2 $$\begin{aligned} \mathcal{T}_s(a_1 f_1 + a_2 f_2) = a_1 \mathcal{T}_s f_1 + a_2 \mathcal{T}_s f_2 \end{aligned}$$

holds for all functions $f_1, f_2 :{\mathbb R}^2 \rightarrow {\mathbb R}$ and all real constants $a_1, a_2 \in {\mathbb R}$. This linearity assumption implies that any special properties that we will derive for the internal representation $L$ will also transfer to any spatial, temporal, or spatio-temporal derivatives of the image data, a property that will be essential regarding early receptive fields, since it implies that different types of image structures will be treated in a similar manner irrespective of what types of linear filters they are captured by.

Furthermore, if we want all image positions $x \in {\mathbb R}^2$ to be treated similarly, such that the visual interpretation of an object remains the same irrespective of its location in the image plane, then it is natural to require the operator $\mathcal{T}_s$ to be *shift invariant* such that

3 $$\begin{aligned} \mathcal{T}_s \left( \mathcal{S}_{\Delta x} f \right) = \mathcal{S}_{\Delta x} \left( \mathcal{T}_s f \right) \end{aligned}$$

holds for all translation vectors $\Delta x \in {\mathbb R}^2$, where $S_{\Delta x}$ denotes the shift (translation) operator defined by $(\mathcal{S}_{\Delta x} f)(x) = f(x-\Delta x)$. Alternatively stated, the operator $\mathcal{T}_s$ can be said to be *homogeneous across space*.^4^

The requirements of linearity and shift invariance together imply that the operator $\mathcal{T}_s$ can be described as a *convolution* transformation^5^ (Hirschmann and Widder 1955)

4 $$\begin{aligned} L(\cdot ;\; s) = T(\cdot ;\; s) * f(\cdot ) \end{aligned}$$

of the form

5 $$\begin{aligned} L(x;\; s) = \int \limits _{\xi \in {\mathbb R}^2} T(\xi ;\; s) \, f(x - \xi ) \, \hbox {d}\xi \end{aligned}$$

for some family of convolution kernels $T(\cdot ;\; s) :{\mathbb R}^2 \rightarrow {\mathbb R}$.

To be able to use tools from functional analysis, we will initially assume that both the original signal $f$ and the family of convolution kernels $T(\cdot ;\; s)$ are in the Banach space $L^2({\mathbb R}^N)$, i.e. that $f \in L^2({\mathbb R}^N)$ and $T(\cdot ;\; s) \in L^2({\mathbb R}^N)$ with the norm

6 $$\begin{aligned} \Vert f \Vert _2^2 = \int \limits _{x \in {\mathbb R}^N} |f(x)|^2 \, \hbox {d}x. \end{aligned}$$

Then, also the intermediate representations $L(\cdot ;\; s)$ will be in the same Banach space and the operators $\mathcal{T}_s$ can be regarded as well defined.

#### Image measurements at different scales

The reduction in the first stage of visual processing to a set of convolution transformations raises the question of what types of convolution kernels $T(\cdot ;\; s)$ could be regarded as natural? Specifically, we may consider convolution kernels with different spatial extent. A convolution kernel having a large spatial support can generally be expected to have the ability to respond to phenomena at coarser scales, whereas a convolution kernel with a small spatial support is generally needed to capture fine-scale phenomena. Hence, it is natural to associate a notion of *scale* with every image measurement. Let us therefore assume that the parameter $s$ represents such a scale attribute and let us assume that this scale parameter should always be nonnegative $s \in {\mathbb R}_+^N$ with the limit case when $s \downarrow 0$ corresponding to an identity operation

7 $$\begin{aligned} \lim _{s \downarrow 0} L(\cdot ;\; s) = \lim _{s \downarrow 0} \mathcal{T}_s f = f. \end{aligned}$$

Hence, the intermediate image representations $L(\cdot ;\, s)$ can be regarded as a family of derived representations parameterized by a scale parameter $s$.^6^

#### Structural requirements on a scale-space representation

*Semigroup and cascade properties* For such image measurements to be properly related *between* different scales, it is natural to require the operators $\mathcal{T}_s$ with their associated convolution kernels $T(\cdot ;\; s)$ to form a *semigroup*

8 $$\begin{aligned} \mathcal{T}_{s_1} \mathcal{T}_{s_2} \!=\! \mathcal{T}_{s_1 + s_2} \Leftrightarrow T(\cdot ;\; s_1) * T(\cdot ;\; s_2) \!=\! T(\cdot ;\; s_1+s_2). \end{aligned}$$

Then, the transformation between any two different and ordered^7^ scale levels $s_1$ and $s_2$ with $s_2 \ge s_1$ will obey the *cascade property*

9 $$\begin{aligned} L(\cdot ;\; s_2)&= T(\cdot ;\; s_2-s_1) * T(\cdot ;\; s_1) * f \nonumber \\&= T(\cdot ;\; s_2-s_1) * L(\cdot ;\; s_1) \end{aligned}$$

i.e., a similar type of transformation as from the original image data $f$. An image representation having these properties is referred to as a *multi-scale representation*.

*Self-similarity* Regarding the choice of convolution kernels to be used for computing a multi-scale representation, it is natural to require them to be *self-similar* over scale (*scale invariant*) in the sense that each kernel $T(\cdot ;\; s)$ can be regarded as a rescaled version of some prototype kernel $\bar{T}(\cdot )$. In the case of a *scalar scale parameter* $s \in {\mathbb R}_+$, such a condition can be expressed as

10 $$\begin{aligned} T(x;\; s) = \frac{1}{\varphi (s)} \bar{T}\left( \frac{x}{\varphi (s)}\right) \end{aligned}$$

with $\varphi (s)$ denoting a monotonously increasing transformation of the scale parameter $s$. For the case of a *multi-dimensional scale parameter* $s \in {\mathbb R}_+^N$, the requirement of self-similarity over scale can be generalized into

11 $$\begin{aligned} T(x;\; s) = \frac{1}{|\det \varphi (s)|} \bar{T}(\varphi (s)^{-1} x) \end{aligned}$$

where $\varphi (s)$ now denotes a non-singular $2 \times 2$-dimensional matrix regarding a 2D image domain and $\varphi (s)^{-1}$ its inverse. With this definition, a multi-scale representation with a scalar scale parameter $s \in {\mathbb R}_+$ will be based on uniform rescalings of the prototype kernel, whereas a multi-scale representation based on a multi-dimensional scale parameter might also allow for rotations as well as non-uniform affine deformations of the prototype kernel.

Together, the requirements of a semigroup structure and self-similarity over scales imply that the parameter $s$ gets both a (i) *qualitative* interpretation of the notion of scale in terms of an abstract *ordering relation* due to the cascade property in Eq. (9) and (ii) a *quantitative* interpretation of scale, in terms of the *scale-dependent spatial transformations* in Eqs. (10) and (11). When these conditions are simultaneously satisfied, we say that the intermediate representation $L(\cdot ;\; s)$ constitutes a candidate for being regarded as a (weak) *scale-space representation*.

*Infinitesimal generator* For theoretical analysis, it is preferable if the scale parameter $s$ can be treated as a continuous parameter and if image representations at adjacent scales can be related by partial differential equations. Such relations can be expressed if the semigroup possesses an *infinitesimal generator* (Hille and Phillips 1957; Pazy 1983)

12 $$\begin{aligned} \mathcal{B} L = \lim _{h \downarrow 0} \frac{T(\cdot ;\; h) * f - f}{h} \end{aligned}$$

and imply that the image representations at adjacent scales can be related by an evolution equation of the form

13 $$\begin{aligned} \partial _s L(x;\; s) = (\mathcal{B} L)(x;\; s) \end{aligned}$$

where we would preferably like the operator $\mathcal{B}$ to be a partial differential operator. The infinitesimal generator is the natural correspondence to a derivative operator for semigroups.

In Eq. (13), we have for simplicity assumed the scale parameter $s$ to be a scalar (one-dimensional) parameter. For a multi-parameter scale space with a scale parameter of the form $s = (s_1, \dots , s_N)$, an analogous concept can be defined in terms of the *directional derivative of the semigroup* along any *positive direction* $u = (u_1, \dots , u_N)$ in the parameter space

14 $$\begin{aligned} (\mathcal{D}_u L)(x;\; s)&= (\mathcal{B}(u) \, L)(x;\; s) \nonumber \\&= \left( u_1 \mathcal{B}_1 + \cdots + u_N \mathcal{B}_N \right) \, L(x;\; s) \end{aligned}$$

where each $\mathcal{B}_k$ $(k = 1 \ldots N)$ constitutes the infinitesimal generator for the parameter $s_k$ along the unit direction $e_k$ in the $N$-dimensional parameter space

15 $$\begin{aligned} \mathcal{B}_k L = \lim _{h \downarrow 0} \frac{T(\cdot ;\; h \, e_k) * f - f}{h} \end{aligned}$$

and with the notion of a “positive direction” in parameter space similar as in footnote 7.

*Smoothing property: non-enhancement of local extrema* A further requirement on a scale-space representation is that convolution with the scale-space kernel $T(\cdot ;\; s)$ should correspond to a *smoothing transformation* in the sense that coarser-scale representations should be guaranteed to constitute *simplifications* of corresponding finer scale representations and that new image structures must not be created at coarser scales $L(\cdot ;\; s)$ that do not correspond to simplifications of corresponding structures in the original data $f$.

For one-dimensional signals $f :{\mathbb R}\rightarrow {\mathbb R}$, such a condition can be formalized as the requirement that the number of local extrema or equivalently the number of zero-crossings in the data must not increase with scale and is referred to as *non-creation of local extrema* (Lindeberg 1990). For higher-dimensional signals, however, it can be shown that there are no non-trivial linear transformations guaranteed to never increase the number of local extrema in an image (Lifshitz and Pizer 1990; Lindeberg 1990).

For higher-dimensional image data, a particularly useful generalization of this notion is that local extrema must not be enhanced with increasing scale (*non-enhancement of local extrema*). In other words, if at some scale level $s_0$ a point $(x_0;\; s_0)$ is a maximum (minimum) over the spatial domain $x$, i.e., for the mapping $x \mapsto L(x;\; s_0)$, then the derivative with respect to scale at this point must not be positive (negative). For a scale-space representation based on a scalar scale parameter, we should hence require (Lindeberg 1990, 1996):

16 $$\begin{aligned} \partial _s L(x_0;\; s_0)&\le 0 \quad \quad \text{ at } \text{ any } \text{ local } \text{ maximum },\end{aligned}$$

17 $$\begin{aligned} \partial _s L(x_0;\; s_0)&\ge 0 \quad \quad \text{ at } \text{ any } \text{ local } \text{ minimum }. \end{aligned}$$

For a multi-parameter scale space, a corresponding requirement on a scale-space representation is that if a point $(x_0;\; s_0)$ is local maximum (minimum) of the mapping $x \mapsto L(x;\; s_0)$, then for *every positive direction* in the $N$-dimensional parameter space, the directional derivative of the semigroup $(\mathcal{D}_u L)(x;\; s)$ according to Eq. (14) must satisfy (Lindeberg 2011):

18 $$\begin{aligned} (\mathcal{D}_u L)(x_0;\; s_0)&\le 0 \quad \quad \text{ at } \text{ any } \text{ local } \text{ maximum },\end{aligned}$$

19 $$\begin{aligned} (\mathcal{D}_u L)(x_0;\; s_0)&\ge 0 \quad \quad \text{ at } \text{ any } \text{ local } \text{ minimum }. \end{aligned}$$

As will be described later, this condition constitutes a *strong restriction* on what convolution kernels $T(\cdot ;\; s)$ can be regarded as *scale-space kernels*.

*Nonnegativity and normalization* Regarding the convolution kernels $T(\cdot ;\; s)$, it is natural to require that any scale-space kernel should be *nonnegative*

20 $$\begin{aligned} T(x;\; s) \ge 0 \end{aligned}$$

and have *unit mass* (unit $L^1$-norm)

21 $$\begin{aligned} \int \limits _{x \in {\mathbb R}^2} T(x;\; s) \, \hbox {d}x = 1. \end{aligned}$$

Nonnegativity follows from the requirement of non-creation of new zero-crossings with increasing scale for one-dimensional signals. Normalization to unit $L^1$-norm can be derived as a consequence of the requirement of non-enhancement of local extrema.

#### Requirements regarding spatial geometry

*Rotational symmetry* For a multi-scale representation based on a scalar scale parameter $s \in {\mathbb R}_+$, it is natural to require the scale-space kernels to be *rotationally symmetric*

22 $$\begin{aligned} T(x;\; s) = h\left( \sqrt{x_1^2 + x_2^2};\; s\right) \end{aligned}$$

for some one-dimensional function $h(\cdot ;\; s) :{\mathbb R}\rightarrow {\mathbb R}$. Such a symmetry requirement can be motivated by the requirement that in the absence of further information, all spatial directions should be equally treated (isotropy).

For a scale-space representation based on a multi-dimensional scale parameter, one may also consider a weaker requirement of rotational invariance at the level of a family of kernels, for example regarding a set of elongated kernels with different orientations in image space. Then, although the individual kernels in the filter family are not rotationally symmetric as individual filters, a collection or a group of such kernels may nevertheless capture image data of different orientation in a rotationally invariant manner, for example if all image orientations are explicitly represented or if the receptive fields corresponding to different orientations in image space can be related by linear combinations.

*Affine covariance* When considering surface patterns that are being deformed by the perspective transformation from the surface of an object to the image plane, a restriction to rotationally symmetric kernels only will, however, interfere with the image deformations that occur if the viewing direction varies in relation to the surface normal. If we approximate the geometry of an image deformation by the derivative of the perspective mapping and assume that there are no illumination variations, then such an image deformation can be modelled by an *affine transformation*

23 $$\begin{aligned} f' = \mathcal{A} \, f \end{aligned}$$

corresponding to

24 $$\begin{aligned} f'(x') = f(x) \quad \text{ with } \quad x' = A \, x + b \end{aligned}$$

where $A$ is a $2 \times 2$ matrix and $b \in {\mathbb R}^2$ a constant offset. Specifically, we can at any image point regard such an affine transformation as a *local linear approximation of the perspective mapping*.

A natural requirement on an idealized vision system that observes objects whose projections on the image plane are being deformed in different ways depending on the viewing conditions is that the vision system should be able to relate or match the different internal representations of external objects that are acquired under different viewing conditions. Such a requirement is natural to enable a stable interpretation of objects in the world under variations of the orientation of the object relative to the observer, to enable invariance under variations of the viewing direction.

**Fig. 4:**
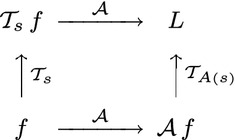
Commutative diagram for scale-space representations computed under affine deformations of image space. Such an affine transformation may, for example, represent a local linear approximation of the projective mapping between two different perspective projections of a surface patch

Hence, if an internal representation $L(\cdot ;\; s)$ of an image pattern $f$ has been computed with a (possibly multi-parameter) scale parameter $s$, we would like the vision system to be able to match this internal representation to the internal representation $L'(\cdot ;\; s')$ of an affine transformed image pattern $f'$ computed with a different (possibly multi-parameter) scale parameter $s'$

25 $$\begin{aligned} L'(x';\; s') = L(x;\; s) \end{aligned}$$

corresponding to

26 $$\begin{aligned} \mathcal{T}_{A(s)} \, \mathcal{A} \, f = \mathcal{A} \, \mathcal{T}_s \, f \end{aligned}$$

as reflected in the commutative diagram in Fig. 4, where $s' = A(s)$ denotes some appropriate transformation of the scale parameter. This requirement is referred to as *affine covariance*. Within the class of linear operators $\mathcal{T}_s$ over a two-dimensional image domain, it is, however, not possible to realize such an affine covariance property within a scale-space concept based on a scalar scale parameter. For two-dimensional image data, such affine covariance can, however, be accomplished within a three-parameter linear scale space.

### Time-dependent image data over a spatio-temporal domain

Regarding spatio-temporal image data $f(x, t)$, which we assume to be defined on a 2+1D spatio-temporal domain ${\mathbb R}^2 \times {\mathbb R}$ with $x = (x_1, x_2)^T$ denoting image space and $t$ denoting time, it is natural to inherit the above-mentioned symmetry requirements expressed for the spatial domain. Hence, corresponding structural requirements as stated in Sects. 2.1.1, 2.1.2, and 2.1.3 should be imposed on a spatio-temporal scale space, with space $x \in {\mathbb R}^2$ replaced by space–time $(x, t) \in {\mathbb R}^2 \times {\mathbb R}$ and with the scale parameter now encompassing also a notion of *temporal scale* $\tau$, such that the multi-dimensional scale parameter $s$ will be of the form $s = (s_1, \ldots , s_N, \tau )$.

#### Additional requirements regarding spatio-temporal geometry

*Galilean covariance* For time-dependent image data, it is natural to also take into explicit account the basic fact that objects may move relative to the observer. Specifically, constant velocity motion

27 $$\begin{aligned} x' = x + v \, t, \end{aligned}$$

where $v = (v_1, v_2)^T$ denotes the image velocity, is referred to as a *Galilean transformation* of space–time

28 $$\begin{aligned} f' = \mathcal{G}_v \, f \end{aligned}$$

corresponding to

29 $$\begin{aligned} f'(x', t') = f(x, t)\quad \text{ with } \quad x' = x + v \, t. \end{aligned}$$

If we assume that the image intensities at corresponding image points remain constant over time $t$ (the constant brightness assumption),^8^ such a Galilean model can be regarded as a *local linear approximation of a more general motion field* $x(t) = (x_1(t), x_2(t))^T$.

**Fig. 5:**
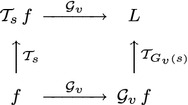
Commutative diagram for a spatio-temporal scale-space representation computed under a Galilean transformation of space–time. Such a constant velocity motion may, for example, represent a local linear approximation of the projected motion field for corresponding image points under relative motions between objects in the world and the visual observer

Analogously to the previously described affine covariance property over a spatial domain, a desirable property of an idealized vision system is that it should be able to compute an internal representation $L(x, t;\; s)$ of a spatio-temporal pattern $f(x, t)$ that can be related or matched to the internal representation of some other spatio-temporal pattern $f'(x', t')$ that moves with a different velocity $v$ relative to the observer. Therefore, we would like to have the ability to relate an internal representation of this pattern $L'(x', t';\; s')$ to the internal representation $L(x, t;\; s)$ of the original pattern for some appropriately transformed scale parameter $s' = G_v(s)$:

30 $$\begin{aligned} L'(x', t';\; s') = L(x, t;\; s) \end{aligned}$$

corresponding to

31 $$\begin{aligned} \mathcal{T}_{G_v(s)} \, \mathcal{G}_v \, f = \mathcal{G}_v \, \mathcal{T}_s \, f \end{aligned}$$

as illustrated in the commutative diagram in Fig. 5. Such a property is referred to as *Galilean covariance*.

Again, within the class of linear transformations $\mathcal{T}_s$, it is not possible to realize such a Galilean covariance property within a spatio-temporal scale concept based solely on a scalar spatial scale parameter $s \in {\mathbb R}$ and a scalar temporal scale parameter $\tau \in {\mathbb R}$. As will be shown later, Galilean covariance can, however, be achieved within a four-parameter linear spatio-temporal scale space.

*Temporal causality* When dealing with time-dependent image data, another structural requirement arises because of the basic fact that the future cannot be accessed. Hence, for any real-time computer vision system or a biological organism that interacts with the world, the convolution kernel must be *time-causal* in the sense that its values must be zero regarding any access to the future

32 $$\begin{aligned} T(x, t;\; s) = 0 \quad \text{ if } \quad t < 0. \end{aligned}$$

When analyzing pre-recorded video data in an off-line situation, we may, however, decide to relax this condition to simplify the computations.

#### Specific constraints regarding a real-time system

*Time recursivity and temporal memory* When dealing with spatio-temporal image data in a real-time setting, we cannot expect the vision system to have direct access to all information from the past, since we cannot assume a computer vision system or a biological organism to store a complete recording of all visual information it has seen.

If we assume that the vision system should compute internal image representations at different temporal scales, the only reasonable approach will therefore be that these computations have to be expressed in terms of computations on some internal temporal buffer $M(x, t)$, which we assume is to be much more condensed than a complete video recording of the past. Such an internal representation is referred to as a *temporal memory*, and the restriction of the set of possible computations to a combination of the current image data $f(x, t)$ with such a compact temporal memory $M(x, t)$ is referred to as *time recursivity*. Specifically, this temporal memory $M(x, t)$ must be updated over time $t$ according to some time-recursive model.

Given the assumption that the vision system should compute an internal scale-space representation $L(x, t; s, \tau )$ at different temporal scales $\tau$ (where we have now changed the notation and separated the spatial scale parameter $s$ from the temporal scale parameter $\tau$), a particularly attractive solution is if this internal representation can also serve as the internal temporal memory $M(x, t;\; \tau )$ for corresponding temporal scales. Let us therefore require that the spatio-temporal scale-space representation $L(x, t; s, \tau )$ should be updated according to a time-recursive evolution equation over scale and time of the form (Lindeberg 2011, section 5.1.3, page 57)

33 $$\begin{aligned}&L(x, t_2; s_2, \tau ) \nonumber \\&\quad = \int \limits _{\xi \in {\mathbb R}^N} \int \limits _{\zeta \ge 0}\! U(x\!-\!\xi , t_2\!-\!t_1;\; s_2 \!-\! s_1, \tau , \zeta ) \nonumber \\&\qquad \qquad \qquad \quad \times L(\xi ,t_1;\; s_1, \zeta ) \, \hbox {d}\zeta \, \hbox {d}\xi \nonumber \\&\qquad + \int \limits _{\xi \in {\mathbb R}^N} \int \limits _{u = t_1}^{t_2} \! B(x\!-\!\xi , t_2\!-\!u;\; s_2, \tau ) \, f(\xi , u) \, \hbox {d}\xi \, \hbox {d}u \end{aligned}$$

for any pair of scale levels $s_2 \ge s_1$ and any two time moments $t_2 \ge t_1$, where

- the kernel $U$ performs the *update on the internal representation* $L$ while simultaneously respecting a cascade property for $L$ over spatial scales $s$ and
- the kernel $h$ *incorporates new information* from the new image data $f(x, t)$ that arrive between $t= t_1$ and $t= t_2$.

*Non-enhancement of local extrema in a time-causal and time-recursive setting* When formalizing the notion of a smoothing operation in a time-causal and time-recursive context, where the internal temporal scale levels $\tau$ are also used as the internal temporal buffer of past information, it turns out to be both useful and necessary to reformulate the requirement of non-enhancement of local extrema in the following way, to take into the fact that at any temporal moment $t_0$, we will have access to image data over space $x$, spatial scales $s$, and temporal scales $\tau$, but no direct access to image data in the future or from the past:

If at some spatial scale $s_0$ and time moment $t_0$ a point $(x_0, \tau _0)$ is a local maximum (minimum) for the mapping $(x, \tau ) \rightarrow L(x, t_0;\; s_0, \tau )$, then for *every positive direction* $u = (u_1, \ldots , u_N, u_{N+1})$ in the $N+1$-dimensional space consisting of the $N$-dimensional spatial scale parameter $s$ complemented by time $t$, the directional derivative $(\mathcal{D}_u L)(x, t;\; s, \tau )$ of the spatio-temporal scale-space representation in this direction $u$ must satisfy (Lindeberg 2011, equations (79)–(80), page 52):

34 $$\begin{aligned}&(\mathcal{D}_u L)(x_0, t_0;\; s_0, \tau _0) \le 0\quad \text{ at } \text{ any } \text{ local } \text{ maximum }, \end{aligned}$$

35 $$\begin{aligned}&(\mathcal{D}_u L)(x_0, t_0;\; s_0, \tau _0) \ge 0 \quad \text{ at } \text{ any } \text{ local } \text{ minimum }.\qquad \end{aligned}$$

This formulation constitutes a generalization of the non-enhancement condition (18) from a regular multi-parameter scale space to a time-recursive multi-parameter scale space. Both of these formulations imply a strong smoothing effect over spatial scales $s$. For a *non-causal* multi-parameter scale-space applied to space–time in a *non-recursive* setting where time $t$ is treated in an essentially similar way as space $x$, non-enhancement of local extrema according to (18) implies a strong evolution property *over temporal scales* $\tau$. The conceptual difference with this *time-recursive formulation* is that the strong temporal smoothing property, as imposed by non-enhancement of local extrema, is instead expressed in terms of the evolution properties *over time* $t$ and not over temporal scales $\tau$.

Notably, this formulation of a temporal evolution property has an interesting interpretation of enforcing a smooth (stabilizing) temporal behavior of the internal representation $L(x, t;\; s, \tau )$ of the surrounding world as the spatio-temporal data $f(x, t)$ varies over time $t$.

### Influence of illumination variations

The above-mentioned symmetry requirements essentially refer to the geometry of space and space–time and its relation to image measurements over non-infinitesimal regions over space or space–time as formalized into the notion of a scale-space representation. Regarding the actual image intensities, these have so far been assumed to be given beforehand.

We may, however, consider different ways of parameterizing the intensity domain. Essentially, any monotonic intensity transformation will preserve the ordering of the intensity values from dark to bright. The perceptual impression of an image may, however, be substantially different after a nonlinear intensity transformation. Hence, one may ask whether we should assume the image data $f$ to be proportional to image irradiance $f \sim I$ (in units of power per unit area), some self-similar power of image irradiance $f \sim I^{\gamma }$ or whether there is a better choice?

*Logarithmic brightness scale* Given the huge range of brightness variations under imaging natural conditions (a range corresponding to a factor of the order of $10^{10}$ between the darkest and brightest cases for human vision), it is natural to represent the image brightness on a *logarithmic scale*:

36 $$\begin{aligned} \begin{array}{l@{\quad }l} f(x) \sim \log I(x) &{} \text{(time-independent } \text{ images) }, \\ f(x, t) \sim \log I(x, t) &{} \text{(spatio-temporal } \text{ image } \text{ data) }. \end{array} \end{aligned}$$

Such a logarithmic scale is also reflected in the construction of most visual sensors (cameras), where aperture steps and exposure times are logarithmically distributed to handle the large range of brightness variations that occur under varying illumination conditions. A local adaptation of the sensitivity of the photoreceptors to an average illumination level can also be seen as implementing an approximation of a logarithmic transformation, provided that both the baseline and the sensitivity regarding deviations from the baseline are adapted in a corresponding manner.

#### Behavior under illumination variations: spatial image data

In this section, we will express properties of a logarithmic brightness scale in relation to a physical illumination model and image measurements in terms of receptive fields.

*Projection model* Consider a *planar perspective camera* model with $X = (X_1, X_2, X_3)^T$ denoting world coordinates with the $X_3$-direction perpendicular to the image plane and with the image coordinates $(x_1, x_2)^T$ for simplicity expressed in units of the focal length $f$, leading to the perspective projection equations (assuming that $X_3 > 0$)

37 $$\begin{aligned} x = (x_1, x_2) ^T = \left( \frac{X_1}{X_3}, \frac{X_2}{X_3} \right) ^T. \end{aligned}$$

Let us furthermore assume that the incoming light is collected by a *thin lens* with diameter $d$.

*Model for image irradiance* Then, given that the image irradiance $I$ is proportional to the surface radiance $R$ along the direction from a point $X$ on the surface toward its projection $X_\mathrm{im} = (x_1, x_2, 1)^T \times f$ on the image plane

38 $$\begin{aligned} I(x) \sim R(X) \end{aligned}$$

or more specifically (Horn 1986, page 208)

39 $$\begin{aligned} I(x) \!=\! R(X) \, \frac{\pi }{4} \left( \frac{d}{f} \right) \cos ^4 \phi (X) \!=\! C_\mathrm{cam}(\tilde{f}) R(X) \cos ^4 \phi (X)\nonumber \\ \end{aligned}$$

with the ratio $\tilde{f} = f/d$ referred to as the *effective f-number*, and with a spatially varying reduction in image intensities toward the periphery of the image (*natural vignetting*) determined by the geometric factor^9^ $\cos ^4 \phi (X)$ with

40 $$\begin{aligned} \cos \phi (X)&= \frac{X_3}{\sqrt{X_1^2 + X_2^2 + X_3^2}} \nonumber \\&= \frac{1}{\sqrt{1+x_1^2+x_2^2}} = \cos \phi (x). \end{aligned}$$

From this expression, it is clear that the proportionality constant in Eq. (38) depends on (i) the internal geometry of the visual sensor as captured by the constant $C_\mathrm{cam}(\tilde{f})$ and (ii) the angle $\phi (x)$ between the viewing direction and the surface normal of the image plane.

*Model for surface reflectance* Let us next assume that the *surface reflectance* $R$ in the direction from the point $X = (X_1, X_2, X_3)^T$ on the surface toward its projection $X_\mathrm{im} = (x_1, x_2, 1)^T$ on the image planed can be modelled as proportional to an *albedo factor* $\rho$ determined by the surface material and the amount of incoming illumination $i$

41 $$\begin{aligned} R(X) \sim \rho (X) \, i(X) \end{aligned}$$

with the implicit assumption that the same amount of light is emitted along all directions from the surface.

This model has a similar behavior as *Lambertian surface model*, with the extension that the surface may be regarded as “gray” by not reflecting all incident light. Please note, however, that this reflectance model constitutes a substantial simplification of the bidirectional reflectance function and does not comprise, e.g., specularities or materials with diffraction grating effects.

For an illumination field that is not determined by a point source only, the entity $i(X)$ can be seen as the integration of the incoming light $i(X, \theta , \varphi )$ from all directions on the northern hemisphere $H$ defined by the spherical coordinates $\theta \in [0, \pi /2]$ and $\varphi \in [0, 2 \pi ]$ relative to the surface normal at $X$ such that

42 $$\begin{aligned} i(X) = \int \limits _H i(X, \theta , \varphi ) \, \cos \theta \, \sin \theta \, \hbox {d}\theta \, \hbox {d}\varphi \end{aligned}$$

where the factor $\cos \theta$ accounts for foreshortening and the factor $\sin \theta$ is the integration measure for spherical coordinates.

*Combined brightness model* By combining the illumination model in Eqs. (39) and (41) with the logarithmic brightness scale in Eq. (36) and by redefining the functions $\rho (X)$ and $i(X)$ such that their values for three-dimensional world coordinates $X$ can be accessed from corresponding projected image coordinates $x$ according to $\rho (x)$ and $i(x)$, we obtain

43 $$\begin{aligned} f(x) \!= \!\log \rho (x) \!+\! \log i(x)\! +\! \log C_\mathrm{cam}(\tilde{f}) \!-\! 2 \log (1 \!+\! x_1^2 \!+\! x_2^2)\nonumber \\ \end{aligned}$$

which provides an explicit model for how the image brightness $f$ depends on

(i) *properties of surfaces of objects* in the world as condensed into the spatially dependent albedo factor $\rho (x)$ with the implicit understanding that this entity may in general refer to different surfaces in the world depending on the viewing direction $(x_1, x_2, 1)^T$ and thus the image position $x = (x_1, x_2)^T$,
(ii) *properties of the illumination field* as reflected in the spatially dependent illumination $i(x)$, which also may refer to the amount of incoming light on different surfaces in the world depending on the value of $x$,
(iii) *geometric properties of the camera* as condensed into a dependency on the effective $f$-number $\tilde{f}$ captured by $C_\mathrm{cam}(\tilde{f})$, and
(iv) a geometric *natural vignetting* effect of the explicit form $V(x) = V(x_1, x_2) = - 2 \log (1 + x_1^2 + x_2^2 )$.

In the following, we shall develop consequences of this image formation model concerning invariance properties to the effective $f$-number and multiplicative illumination transformations, given the specific choice of a logarithmic brightness scale.

*Invariance to the effective f-number* A noteworthy property of the model in Eq. (43) is that if we disregard effects of focal blur (not modelled here), then the influence due to the internal focal distance $f$ and the diameter $d$ of the camera will be *cancelled*, if we differentiate this expression with respect to space $x$

44 $$\begin{aligned} (\partial _{x^{\alpha }} f)(x)&= (\partial _{x_1^{\alpha _1} x_2^{\alpha _2}} f)(x_1, x_2) \nonumber \\&= \partial _{x^{\alpha }} \left( \log \rho (x) \!+\! \log i(x) \!-\! 2 \log (1 \!+\! x_1^2 \!+\! x_2^2 ) \right) \end{aligned}$$

where $\alpha = (\alpha _1, \alpha _2)$ constitutes a multi-index notation. Hence, with a logarithmic brightness scale (and disregarding effects of focal blur), any spatial derivative operator will be *invariant to variations in the effective f-number* (as well as other multiplicative exposure parameters).

*Invariance to multiplicative illumination transformations* Moreover, if we consider image measurements from the same scene using a different illumination field $i'(x)$ proportional to the original illumination field

45 $$\begin{aligned} i'(x) = C_\mathrm{illum} \, i(x), \end{aligned}$$

then it follows that the influence of $C_\mathrm{illum}$

46 $$\begin{aligned} f'(x)&= \log \rho (x) \!+\! \log C_\mathrm{illum} \!+\! \log i(x) \!+\! \log C_\mathrm{cam}(\tilde{f})\nonumber \\&- 2 \log (1 \!+\! x_1^2 \!+\! x_2^2)\!=\! f(x) \!+\!\log C_\mathrm{illum} \end{aligned}$$

will also be cancelled after spatial differentiation

47 $$\begin{aligned} (\partial _{x^{\alpha }} f')(x) = (\partial _{x^{\alpha }} f)(x) \end{aligned}$$

Therefore, with a logarithmic brightness scale, any spatial derivative operator will be *invariant to multiplicative illumination transformations.* The influence of the constant $\log C_\mathrm{illum}$ will also disappear after filtering with a kernel having integral zero, i.e., equal positive and negative contributions.

*Relative measurements of physical entities* Furthermore, regarding, e.g., any first-order derivative $\partial _{x_k}$ with $k$ equal to 1 or 2

48 $$\begin{aligned}&(\partial _{x_k} f)(x_1, x_2) = \frac{(\partial _{x_k} \rho )(x_1, x_2)}{\rho (x_1, x_2)} + \frac{(\partial _{x_k} i)(x_1, x_2)}{i(x_1, x_2)} \nonumber \\&- \frac{4 x_k}{1 + x_1^2 + x_2^2} \end{aligned}$$

the interpretation of this first-order spatial derivative operator is that it responds to *relative variations* of the physical entities surface albedo $\rho (x)$ and the illumination $i(x)$ (where we assume these quantities to always be strictly positive and never becoming equal to zero):

- For a smooth surface with a spatially dependent surface pattern $\rho (X)$, the first term $\partial _{x_k} \rho /\rho$ reflects inherent *relative spatial variations of this surface pattern* as deformed by the perspective projection model in analogy with the affine deformation model (24).
- The second term $\partial _{x_k} i/i$ reflects *relative spatial variations in the illumination field* $i$ as arising from the interaction between the external illumination field $i(X, \theta (X), \varphi (X))$ and the local surface geometry $(\theta (X), \varphi (X))$ at every surface point $X$ according to (42).
- The third term $(\partial _{x_k} V)(x) = (\partial _{x_k} V)(x_1, x_2) = 4 x_k/(1 + x_1^2 + x_2^2)$ constitutes a *geometric bias due to vignetting effects* inherent to the camera. (Please note that the image coordinates in this treatment are expressed in units of the focal length with $|x| = \sqrt{x_1^2 + x_2^2} \ll 1$ in the central field of view.) This term will disappear for a spherical camera geometry.

If the surface albedo $\rho (x)$ and the illumination field $i(x)$ are also measured on a logarithmic scale, then the algebraic relationship between derivatives of image intensity $f$ and derivatives of the physical entities $\rho (x)$ and $i(x)$ will be simple also for any order of differentiation

49 $$\begin{aligned} (\partial _{x^{\alpha }} f')(x)&= \partial _{x^{\alpha }} \left( \log \rho (x) \right) \nonumber \\&+ \partial _{x^{\alpha }} \left( \log i(x) \right) + \partial _{x^{\alpha }} \left( \log V(x) \right) \!. \end{aligned}$$

*Invariance properties of spatial receptive fields involving spatial derivatives* There is an interesting relationship between the cancelling of multiplicative illumination transformations in Eq. (44) and image measurements in terms of receptive fields. If we consider the derived internal scale-space representation $L$ of a signal $f$ and compute any spatial derivative of this representation according to

50 $$\begin{aligned} \partial _{x^{\alpha }} L&= \partial _{x^{\alpha }} \mathcal{T}_s \, f = \mathcal{T}_s \, \partial _{x^{\alpha }} \, f \nonumber \\&= \mathcal{T}_s \, \partial _{x^{\alpha }} \left( \log \rho + \log i + \log V\right) \end{aligned}$$

then it follows that *the effect of any multiplicative illumination transformation will be invisible to image measurements in terms of receptive fields* $\partial _{x^{\alpha }} \mathcal{T}_s$ *that involve spatial derivatives*. Similarly, besides effects of focal blur, the intensity dependency due to variations of the effective $f$-number $\tilde{f}$ will also cancel. Hence, with a logarithmic brightness scale, image measurements in terms of receptive fields that involve spatial derivatives (or more generally any receptive field with its integral equal to zero) will be *invariant under multiplicative illumination transformations and exposure conditions*, with the latter corresponding to variations of the exposure time, the aperture and the ISO number of the sensor in a digital camera, or the diameter of the pupil and the photosensitivity of the photoreceptors in biological vision. The remaining response is a superposition of relative variations in surface patterns and illumination variations, with a position-dependent bias due to the vignetting effect.

It should be noted, however, that some care is needed concerning the *differentiability properties* of the image data. For images acquired from a natural world, there will in general be discontinuities in image brightness $f$, due to discontinuities in depth, surface orientation, illumination, or the albedo of the surface patterns, which implies that we would generally expect to obtain strong spikes in the output if plain derivative operators would be applied to natural image data. The use of *receptive field-based derivative operations*, however, regularizes this problem. For the families of smoothing kernels $T(\cdot ;\, s)$ that can be derived from the requirement of non-enhancement of local extrema, it can be shown that the scale-space representation $L(\cdot ;\, s)$ will indeed become *infinitely differentiable* after any non-infinitesimal amount of smoothing $s > 0$ if we assume bounded brightness data $|f(x)| < C$. Hence, the output from the receptive field-based derivative operators $\partial _{x^{\alpha }} T(\cdot ;\, s)$ will always be well defined and the validity of the results in Eqs. (44) and (50) can be formally established with $(\partial _{x^{\alpha }} f)(x)$ replaced by $(\partial _{x^{\alpha }} L)(x;\; s)$:

51 $$\begin{aligned} \partial _{x^{\alpha }} L = \partial _{x^{\alpha }} \mathcal{T}_s \, \left( \log \rho + \log i + \log V \right) \!. \end{aligned}$$

Indeed, the notion of receptive field-based derivative approximations can be regarded as *necessary* to make these computations of image derivatives valid. The assumption of linearity as a basic scale-space axiom in Eq. (2) can also be motivated from the form of this expression, by making it possible to interpret the receptive field responses as a linear superposition of relative variations in surface patterns and relative variations in the illumination field. Such an interpretation would not be possible if the smoothing operator $\mathcal{T}_s$ would be nonlinear.

*Scale-space properties of receptive field measurements involving spatial derivatives* Due to the linearity property, receptive field measurements involving spatial derivatives $\partial _{x^{\alpha }} L$ will possess essentially similar scale-space properties over scales as possessed by the zero-order scale-space representation $L$ of the original illumination pattern $f$ as described in Sect. 2.1.3, with the main difference that the limit case in Eq. (7) when the scale parameter $s$ tends to zero has to be replaced by

52 $$\begin{aligned} \lim _{s \downarrow 0} L_{x^{\alpha }}(\cdot ;\; s) = \lim _{s \downarrow 0} \partial _{x^{\alpha }} \mathcal{T}_s f = \partial _{x^{\alpha }} f \end{aligned}$$

provided that the image data $f$ have sufficient differentiability properties.

#### Behavior under illumination variations: spatio-temporal image data

*Invariance properties of spatial receptive fields involving spatio-temporal derivatives* For spatio-temporal image data, the corresponding image formation model becomes

53 $$\begin{aligned} f(x, t)&= \log \rho (x, t) + \log i(x, t) + \log C_\mathrm{cam}(\tilde{f}(t)) \nonumber \\&- 2 \log (1 + x_1^2 + x_2^2) \end{aligned}$$

if we allow the effective $f$-number to depend on time $t$. If we measure such spatio-temporal image data using a spatio-temporal receptive field with a spatio-temporal scale parameter $s = (s_1, \ldots , s_N, \tau )$ that involves integration over both space $x$ and time $t$, and if we differentiate such a representation with respect to both space and time

54 $$\begin{aligned} \partial _{x^{\alpha } t^{\beta }} L&= \partial _{x^{\alpha } t^{\beta }} \left( \mathcal{T}_s \, f \right) = \left( \partial _{x^{\alpha } t^{\beta }} \mathcal{T}_s \right) \, f = \mathcal{T}_s \, \partial _{x^{\alpha } t^{\beta }} \, f \nonumber \\&= \mathcal{T}_s \, \partial _{x^{\alpha } t^{\beta }} \left( \log \rho + \log i \right) \!, \end{aligned}$$

then it follows that the influence of the possibly time-dependent effective $f$-number will be cancelled after any spatial derivative operation with $|\alpha | > 0$ (and so will the influence be of any other possibly time-dependent multiplicative exposure control mechanism).

Regarding temporal derivatives, it follows that the influence of the vignetting effect $V(x)$ will be cancelled by any temporal derivative operator with $\beta \ge 0$. The temporal derivative operator will also suppress the effect of any other solely spatial illumination variation.

*Galilean covariant temporal derivative concept* When considering temporal derivatives of spatio-temporal data computed for an object that moves with image velocity $v = (v_1, v_2)^T$ relative to the observer, it is natural to consider *velocity-adapted temporal derivatives* $\partial _{\bar{t}}$ along the direction of motion according to

55 $$\begin{aligned} \partial _{\bar{t}} = \partial _t + v^T \nabla _x = \partial _t +v_1 \, \partial _{x_1} + v_2 \, \partial _{x_2} \end{aligned}$$

so as to obtain a temporal derivative concept that commutes with Galilean transformations. Such velocity-adapted temporal derivatives make it possible to compute *Galilean covariant image representations based on receptive fields involving temporal derivatives*, in analogy with the previous treatment of Galilean covariance in connection with Eq. (31).

#### Summary regarding intensity and illumination variations

To summarize, this analysis shows that with image intensities parameterized on a logarithmic brightness scale and provided that the smoothing operation $\mathcal{T}_s$ has sufficient regularizing properties to make the computation of image derivatives well defined, *receptive field responses in terms of spatial and spatio-temporal derivatives have a direct physical interpretation* as the superposition of

- relative variations in the albedo of the observed surface patterns corresponding to the term $\partial _{x^{\alpha } t^{\beta }} \left( \mathcal{T}_s \, \log \rho (x) \right)$ in (54), and
- relative variations in the illumination field corresponding to the term $\partial _{x^{\alpha } t^{\beta }} \left( \mathcal{T}_s \, \log i(x) \right)$ in (54)

with a geometric bias caused by vignetting effects that disappears for temporal derivatives with $\beta > 0$. Moreover, such receptive field measurements are *invariant under multiplicative illumination transformations* as well as other multiplicative exposure control mechanisms.

## Spatial domain with pure intensity information

We shall now describe how the structural requirements on an idealized vision system as formulated in Sect. 2.1 restrict the class of possible image operations at the first stages of visual processing. For image data $f :{\mathbb R}^2 \rightarrow {\mathbb R}$ defined over a *two-dimensional spatial domain*, let us assume that the first stage of visual processing as represented by the operator $\mathcal{T}_s$ should be (i) *linear*, (ii) *shift invariant*, and (iii) obey a *semigroup structure over spatial scales* $s$, where we also have to assume (iv) certain *regularity properties* of the semigroup $\mathcal{T}_s$ *over scale* $s$ in terms of Sobolev norms^10^ to guarantee sufficient differentiability properties with respect to space $x \in {\mathbb R}^2$ and scale $s$. Let us furthermore require (v) *non-enhancement of local extrema* to hold for *any* smooth image function $f \in C^{\infty }({\mathbb R}^2) \cap L^1({\mathbb R}^2)$.

Then, it can be shown (Lindeberg 2011, Theorem 5, page 42) that these conditions together imply that the scale-space family $L$ must satisfy a diffusion equation of the form

56 $$\begin{aligned} \partial _s L = \frac{1}{2} \nabla _x^T \left( \varSigma _0 \nabla _x L \right) - \delta _0^T \nabla _x L \end{aligned}$$

with the notation $\nabla _x = (\partial _{x_1}, \partial _{x_2})^T$ for the gradient operator, and with initial condition $L(\cdot ;\; 0) = f(\cdot )$ for some positive semi-definite $2 \times 2$ covariance matrix $\varSigma _0$ and for some 2D vector $\delta _0$, where the covariance matrix $\varSigma _0$ describes the shape of the underlying smoothing kernel and the vector $\delta _0$ describes the spatial offset or the drift velocity of a non-symmetric smoothing kernel. In terms of convolution transformations, this scale space can equivalently be constructed by convolution with *affine and translated Gaussian kernels*

57 $$\begin{aligned} g(x;\; \varSigma _s, \delta _s) = \frac{1}{2 \pi \sqrt{\det \varSigma _s}} \, \hbox {e}^{- {(x - \delta _s)^T \varSigma _s^{-1} (x - \delta _s)}/{2}} \end{aligned}$$

which for a given $\varSigma _s = s \, \varSigma _0$ and a given $\delta _s = s \, \delta _0$ satisfy the diffusion equation (56).

### Gaussian receptive fields

**Fig. 6:**
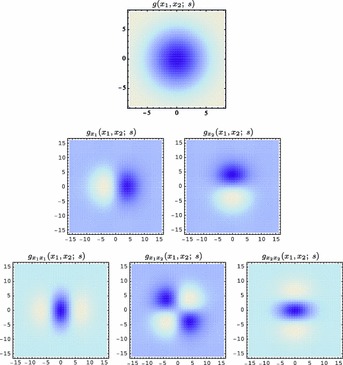
Spatial receptive fields formed by the 2D Gaussian kernel with its partial derivatives up to order two. The corresponding family of receptive fields is closed under translations, rotations, and scaling transformations, meaning that if the underlying image is subject to a set of such image transformations, then it will always be possible to find some possibly other receptive field such that the receptive field responses of the original image and the transformed image can be matched

If we require the corresponding convolution kernels to be rotationally symmetric, then it follows that they will be Gaussians

58 $$\begin{aligned} T(x;\, s) = g(x;\; s) = \frac{1}{2 \pi s} \, e^{-x^T x/2 s} = \frac{1}{2 \pi s} \, e^{-(x_1^2 + x_2^2)/2 s}\nonumber \\ \end{aligned}$$

with corresponding *Gaussian derivative operators*

59 $$\begin{aligned} (\partial _{x^{\alpha }} g)(x;\; s)&= (\partial _{x_1^{\alpha _1} x_2^{\alpha _2}} g)(x_1, x_2;\; s) \nonumber \\&= (\partial _{x_1^{\alpha _1}} \bar{g})(x_1;\; s) \, (\partial _{x_2^{\alpha _2}} \bar{g})(x_2;\; s) \end{aligned}$$

(with $\alpha = (\alpha _1, \alpha _2)$ where $\alpha _1$ and $\alpha _2$ denote the order of differentiation in the $x_1$- and $x_2$-directions, respectively) as shown in Fig. 6 with the corresponding one-dimensional Gaussian kernel and its Gaussian derivatives of the form:

60 $$\begin{aligned}&\bar{g}(x_1;\; s) = \frac{1}{\sqrt{2 \pi s}} e^{-x_1^2/2s}, \end{aligned}$$

61 $$\begin{aligned}&\bar{g}_{x_1}(x_1;\; s) = - \frac{x_1}{s} \bar{g}(x_1;\; s) = - \frac{x_1}{\sqrt{2 \pi } s^{3/2}} e^{-x_1^2/2s}, \end{aligned}$$

62 $$\begin{aligned}&\bar{g}_{x_1x_1}(x_1;\; s) = \frac{(x_1^2 - s)}{s^2} \bar{g}(x_1;\; s) = \frac{(x_1^2 - s)}{\sqrt{2 \pi } s^{5/2}} e^{-x_1^2/2s}.\nonumber \\ \end{aligned}$$

Such Gaussian functions have been previously used for modelling biological vision by Young (1987), who has shown that there are receptive fields in the striate cortex that can be well modelled by Gaussian derivatives up to order four. More generally, these Gaussian derivative operators or approximations thereof can be used as a *general basis* for expressing image operations such as feature detection, feature classification, surface shape, image matching, and image-based recognition (Iijima 1962; Witkin 1983; Koenderink 1984; Koenderink and Doorn 1992; Lindeberg 1994a, b, 1998a, b, 2008; Florack 1997; Schiele and Crowley 1996, 2000; Lowe 1999, 2004; Chomat et al. 2000; ter Haar Romeny 2003; Linde and Lindeberg 2004, 2012; Bay et al. 2008). Specifically, this receptive field model makes it possible to compute *scale-invariant image features and image descriptors* (Crowley 1981; Crowley and Stern 1984; Lindeberg 1998a, b, 1999, 2013; Lowe 1999, 2004; Schiele and Crowley 2000; Chomat et al. 2000; Bay et al. 2008). Other necessity results concerning Gaussian and Gaussian derivative kernels have been presented by Iijima (1962), Koenderink (1984), Koenderink and Doorn (1992), Babaud et al. (1986), Yuille and Poggio (1986), Lindeberg (1990, 1994b, 1996), and Florack and Haar Romeny (1992).

### Affine-adapted Gaussian receptive fields

If we relax the requirement of rotational symmetry into a requirement of mirror symmetry through the origin, then it follows that the convolution kernels must instead be *affine Gaussian kernels*

63 $$\begin{aligned} T(x;\; s) = g(x;\; \varSigma ) = \frac{1}{2 \pi \sqrt{\det \varSigma }} e^{-x^T \varSigma ^{-1} x/2} \end{aligned}$$

where $\varSigma$ denotes any symmetric positive semi-definite $2 \times 2$ matrix. This affine scale-space concept is *closed* under affine transformations, meaning that if we for two affine-related images

64 $$\begin{aligned} f_L(\xi ) = f_R(\eta ) \quad \text{ where } \quad \eta = A \, \xi + b \end{aligned}$$

define corresponding scale-space representations according to

65 $$\begin{aligned} L(\cdot ;\; \varSigma _L)&= g(\cdot ;\; \varSigma _L) * f_L(\cdot ) \nonumber \\ R(\cdot ;\; \varSigma _R)&= g(\cdot ;\; \varSigma _R) * f_R(\cdot ), \end{aligned}$$

then these scale-space representations will be related according to (Lindeberg 1994b; Lindeberg and Gårding 1997)

66 $$\begin{aligned} L(x;\; \varSigma _L) = R(y;\; \varSigma _R) \end{aligned}$$

where

67 $$\begin{aligned} \varSigma _R = A \, \varSigma _L \, A^T \quad \text{ and } \quad y = A \, x + b. \end{aligned}$$

In other words, given that an image $f_L$ is affine transformed into an image $f_R$, it will always be possible to find a transformation between the scale parameters $s_L$ and $s_R$ in the two domains that make it possible to match the corresponding derived internal representations $L(\cdot ;\; s_L)$ and $R(\cdot ;\, s_R)$.

**Fig. 7:**
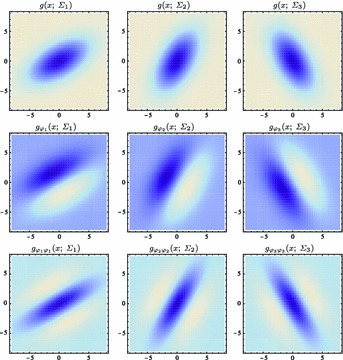
Spatial receptive fields formed by affine Gaussian kernels and directional derivatives of these, here using three different covariance matrices $\varSigma _1$, $\varSigma _2$, and $\varSigma _3$ corresponding to the directions $\theta _1 = \pi /6$, $\theta _2 = \pi /3$, and $\theta _3 = 2\pi /3$ of the major eigendirection of the covariance matrix and with first- and second-order directional derivatives computed in the corresponding orthogonal directions $\varphi _1$, $\varphi _2$, and $\varphi _3$. The corresponding family of receptive fields is closed under general affine transformations of the spatial domain, including translations, rotations, scaling transformations, and perspective foreshortening (although this figure only illustrates variabilities in the orientation of the filter, thereby disregarding variations in both size and degree of elongation)

Figure 7 shows a few examples of such kernels in different directions with the covariance matrix parameterized according to

68 $$\begin{aligned} \varSigma&= \left( \begin{array}{c@{\quad }c@{\quad }c} \lambda _1 \cos ^2 \theta + \lambda _2 \sin ^2 \theta \quad &{} (\lambda _1 - \lambda _2) \cos \theta \, \sin \theta \\ (\lambda _1 - \lambda _2) \cos \theta \, \sin \theta \quad &{} \lambda _1 \sin ^2 \theta + \lambda _2 \cos ^2 \theta \end{array} \right) \end{aligned}$$

with $\lambda _1$ and $\lambda _2$ denoting the eigenvalues and $\theta$ the orientation. Directional derivatives of these kernels can in turn be obtained from linear combinations of partial derivative operators according to

69 $$\begin{aligned} \partial _{\varphi ^m} L&= (\cos \varphi \, \partial _{x_1} + \sin \varphi \, \partial _{x_2})^m L \nonumber \\&= \sum _{k=0}^m \left( \begin{array}{c} m \\ k \end{array} \right) \cos ^k \varphi \, \sin ^{m-k} \varphi \, L_{x_1^k x_2^{m-k}}. \end{aligned}$$

This “steerability” property is a basic consequence of the definition of directional derivatives and has been popularized for image processing applications by Freeman and Adelson (1991).

With respect to biological vision, the affine Gaussian kernels as well as directional derivatives of these can be used for modelling receptive fields that are oriented in the spatial domain, as will be described in connection with Eq. (111) in Sect. 6. For computational vision, they can be used for computing *affine invariant image features and image descriptors* for, e.g., cues to surface shape, image-based matching, and recognition (Lindeberg 1994b; Lindeberg and Gårding 1997; Baumberg 2000; Mikolajczyk and Schmid 2004; Tuytelaars and Gool 2004; Lazebnik et al. 2005; Rothganger et al. 2006).

**Fig. 8:**
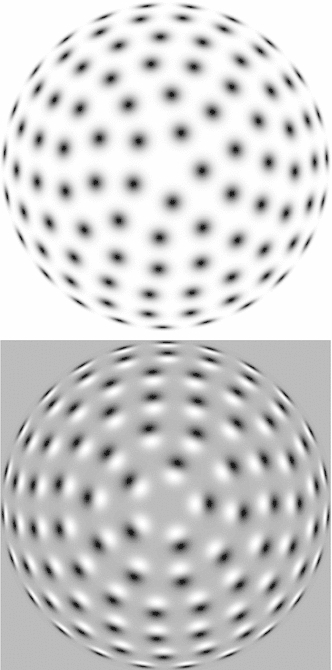
Distributions of affine Gaussian receptive fields corresponding to a uniform distribution on a hemisphere regarding (*top*) zero-order smoothing kernels and (*bottom*) first-order derivatives. In the most idealized version of the theory, one can think of all affine receptive fields as being present at any position in the image domain. When restricted to a limited number of receptive fields in an actual implementation, there is also an issue of distributing a fixed number of receptive fields over the spatial coordinates $x$ and the filter parameters $\varSigma$

Figure 8 shows the distributions of affine receptive fields of different orientations and degrees of orientation as they arise from local linearizations of a perspective projection model if we assume that the set of surface directions in the world is on average uniformly distributed in the world and if the distributions of the local surface patterns on these object surfaces are in turn without dominant directional bias and uncoupled to the orientations of the local surface patches. In our idealized model of receptive fields, all these receptive fields can be thought of as being present at every position in image space and corresponding to a uniform distribution on a hemisphere.

### Necessity of derived receptive fields in terms of derivatives

Due to the linearity of the differential equation (57), which has been derived by necessity from the structural requirements, it follows that also the result of applying a linear operator $\mathcal{D}$ to the solution $L$ will satisfy the differential equation, however, with a different initial condition

70 $$\begin{aligned} \lim _{s \downarrow 0} (\mathcal{D} L)(\cdot ;\; s) = \mathcal{D} f. \end{aligned}$$

The result of applying a linear operator $\mathcal{D}$ to the scale-space representation $L$ will therefore satisfy the above-mentioned structural requirements of linearity, shift invariance, the weaker form of rotational invariance at the group level^11^ and non-enhancement of local extrema, with the semigroup structure (8) replaced by the cascade property

71 $$\begin{aligned} (\mathcal{D} L)(\cdot ;\; s_2) = T(\cdot ;\; s_2-s_1) * (\mathcal{D} L)(\cdot ;\; s_1). \end{aligned}$$

Then, one may ask whether any linear operator $\mathcal{D}$ would be reasonable? From the requirement of scale invariance, however, if follows that the operator $\mathcal{D}$ must not be allowed to have non-infinitesimal support, since a non-infinitesimal support $s_0 > 0$ would violate the requirement of self-similarity over scale (10) and it would not be possible to perform image measurements at a scale level lower than $s_0$. Thus, any receptive field operator derived from the scale-space representation in a manner compatible with the structural arguments must correspond to local derivatives. In the illustrations above, partial derivatives and directional derivatives up to order two have been shown.

For directional derivatives that have been derived from elongated kernels whose underlying zero-order convolution kernels are not rotationally symmetric, it should be noted that we have aligned the directions of the directional derivative operators to the orientations of the underlying kernels. A structural motivation for making such an alignment can be obtained from a requirement of a weaker form of rotational symmetry at the group level. If we would like the family of receptive fields to be rotationally symmetric as a group, then it is natural to require the directional derivative operators to be transformed in a similar way as the underlying kernels.

## Spatial domain with color information

To define a corresponding scale-space concept for color images, the simplest approach would be by computing a Gaussian scale-space representation for each color channel individually. Since the values of the color channels will usually by highly correlated, it is, however, preferable to *decorrelate* the dependencies by computing a color-opponent representation. Such a representation is also in good agreement with human vision, where a separation into red/green and yellow/blue color-opponent channels takes place at an early stage in the visual pathways.

### Gaussian color-opponent receptive fields

Given three RGB channels obtained from a color sensor, consider a color-opponent transformation of the form (Hall et al. 2000)

72 $$\begin{aligned} \left( \begin{array}{c} f\\ c^{(1)}\\ c^{(2)} \end{array} \right) = \left( \begin{array}{c@{\quad }c@{\quad }c} \tfrac{1}{3} &{} \tfrac{1}{3} &{} \tfrac{1}{3} \\ \tfrac{1}{2} &{} - \tfrac{1}{2} &{} 0 \\ \tfrac{1}{2} &{} \tfrac{1}{2} &{} -1 \\ \end{array} \right) \left( \begin{array}{c} R \\ G\\ B \end{array} \right) \end{aligned}$$

where yellow is approximated by the average of the $R$ and $G$ channels $Y = (R + G)/2$ and $f = (R + G + B)/3$ is defined as a channel of pure intensity information. Then, a *Gaussian color-opponent scale-space representation* $(C^{(1)}, C^{(2)})$ can be defined by applying Gaussian convolution to the color-opponent channels $(c^{(1)}, c^{(2)})^T$:

73 $$\begin{aligned} C^{(1)}(\cdot , \cdot ;\; t)&= g(\cdot , \cdot ;\; t) * c^{(1)}(\cdot ), \end{aligned}$$

74 $$\begin{aligned} C^{(2)}(\cdot , \cdot ;\; t)&= g(\cdot , \cdot ;\; t) * c^{(2)}(\cdot ). \end{aligned}$$

**Fig. 9:**
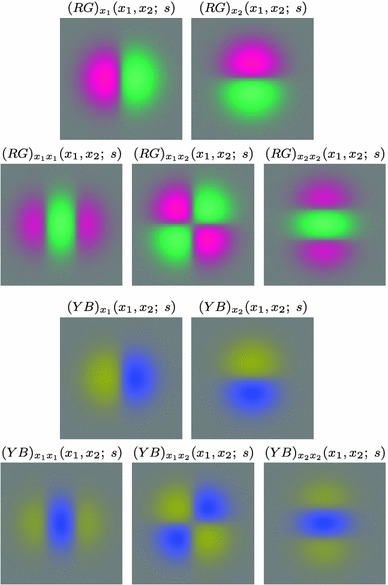
Spatio-chromatic receptive fields corresponding to the application of Gaussian derivative operators up to order two to *red*/*green*, and *yellow*/*blue* color-opponent channels, respectively

**Fig. 10:**
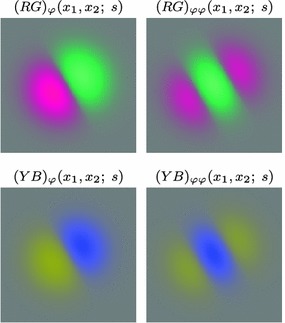
Spatio-chromatic receptive fields corresponding to the application of Gaussian directional derivatives up to order two along the direction $\varphi = \pi /6$ to *red*/*green* and *yellow*/*blue* color-opponent channels, respectively

Figure 9 shows equivalent spatio-chromatic receptive fields corresponding to the application of Gaussian derivative operators according to (59) to such color-opponent channels. Figure 10 shows examples of applying corresponding directional derivatives according to (69).

In Hall et al. (2000), Linde and Lindeberg (2004, 2012), and Sande et al. (2010), it is shown how such spatio-chromatic receptive fields in combination with regular spatial receptive fields can constitute an effective basis for object recognition.

Another type of Gaussian color model has been proposed by Koenderink and later used by Geusebroek and his co-workers (Burghouts and Geusebroek 2009) with receptive fields defined over the spectrum of wavelengths in the color spectrum, corresponding to zero-, first-, and second-order derivatives with respect to wavelength.

## Spatio-temporal image data

### Non-causal spatio-temporal receptive fields

Let us first apply a similar way of reasoning as in Sect. 3 with space $x \in {\mathbb R}^2$ replaced by space–time $(x, t)^T \in {\mathbb R}^2 \times {\mathbb R}$ and disregarding temporal causality, thereby allowing unlimited access to information over both space and time. Given image data $f :{\mathbb R}^2 \times {\mathbb R}\rightarrow {\mathbb R}$ defined over a 2+1D spatio-temporal domain, let us therefore again assume that the first stage of visual processing as represented by the operator $\mathcal{T}_s$ should be (i) *linear*, (ii) *shift invariant*, and (iii) obey a *semigroup structure over both spatial and temporal scales* $s$, where we also assume (iv) certain *regularity properties* of the semigroup $\mathcal{T}_s$ *over scale* $s$ in terms of Sobolev norms^12^ to guarantee sufficient differentiability properties with respect to space $x$, time $t$ and spatio-temporal scales $s$. Let us furthermore require (iv) *non-enhancement of local extrema* to hold for *any* smooth image function $f \in C^{\infty }({\mathbb R}^2 \times {\mathbb R}) \cap L^1({\mathbb R}^2 \times {\mathbb R})$ and for any positive scale direction $s$.

Then, it follows from (Lindeberg (2011), Theorem 5, page 42) that the scale-space representation over a 2+1D spatio-temporal domain must satisfy

75 $$\begin{aligned} \partial _s L = \frac{1}{2} \, \nabla _{(x,t)}^T \left( \varSigma _0 \nabla _{(x,t)} L \right) - \delta _0^T \, \nabla _{(x,t)} L \end{aligned}$$

for some $3 \times 3$ covariance matrix $\varSigma _0$ and some 3D vector $\delta _0$ with $\nabla _{(x, t)} = (\partial _{x_1}, \partial _{x_2}, \partial _t)^T$.

**Fig. 11:**
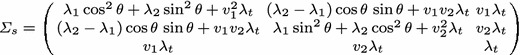
Parameterization of the spatio-temporal covariance matrix for the Gaussian spatio-temporal scale space in terms of the spatial eigenvalues $\lambda _1$ and $\lambda _2$ with the associated orientation $\theta$ for the purely spatial covariance matrix, the image velocity $v = (v_1, v_2)^T$, and the amount of temporal smoothing $\lambda _t$

In terms of convolution kernels, the zero-order receptive fields will then be *spatio-temporal Gaussian kernels*

76 $$\begin{aligned} g(p;\; \varSigma _s, \delta _s) = \frac{1}{(2 \pi )^{3/2} \sqrt{\hbox {det} \varSigma _s}} \, e^{- {(p - \delta _s)^T \varSigma _s^{-1} (p - \delta _s)}/{2s}}\nonumber \\ \end{aligned}$$

with $$p = (x, t)^T = (x_1, x_2, t)^T$$,

77 $$\begin{aligned} \varSigma _s&= \{3 \times 3\; \text {matrix as shown in Fig. 11} \} \end{aligned}$$

78 $$\begin{aligned} \delta _s&= \left( \begin{array}{c} v_1 t \\ v_2 t \\ \delta \end{array} \right) \end{aligned}$$

where (i) $\lambda _1$, $\lambda _2$, and $\theta$ determine the *spatial extent*, (ii) $\lambda _t$ determines the *temporal extent*, (iii) $v = (v_1, v_2)^T$ denotes the *image velocity* and (iv) $\delta$ represents a *temporal delay* and corresponding to a coupling between the spatial and temporal dimensions of the form

79 $$\begin{aligned} g(x, t;\; s, \tau ;\; \varSigma , v) = g(x - v t;\; s;\; \varSigma ) \, \bar{g}(t;\; \tau , \delta ) \end{aligned}$$

where $\bar{g}(t;\; \tau , \delta )$ denotes a one-dimensional Gaussian kernel over time with temporal extent $\tau$ and temporal delay $\delta$. From the corresponding *Gaussian spatio-temporal scale space*

80 $$\begin{aligned} L(x, t;\; \varSigma _\mathrm{space}, v, \tau ) \!=\! (g(\cdot , \cdot ;\; \varSigma _\mathrm{space}, v, \tau ) * f(\cdot , \cdot ))(x, t)\nonumber \\ \end{aligned}$$

spatio-temporal derivatives can then be defined according to

81 $$\begin{aligned} L_{x^{\alpha } t^{\beta }}(x, t;\; \varSigma _\mathrm{space}, v, \tau ) = (\partial _{x^{\alpha } t^{\beta }} L)(x, t;\; \varSigma _\mathrm{space}, v, \tau )\nonumber \\ \end{aligned}$$

with corresponding *velocity-adapted temporal derivatives*

82 $$\begin{aligned} \partial _{\bar{t}} = v^T \nabla _x + \partial _t = v_1 \, \partial _{x_1} + v_2 \, \partial _{x_2} + \partial _t \end{aligned}$$

as illustrated in Figs. 12 and 13 for the case of a 1+1D space–time. Motivated by the requirement of Galilean covariance, it is natural to align the directions $v$ in space–time for which these velocity-adapted spatio-temporal derivatives are computed to the velocity values used in the underlying zero-order spatio-temporal kernels, since the resulting velocity-adapted spatio-temporal derivatives will then be Galilean covariant. Such receptive fields or approximations thereof can be used for modelling spatio-temporal receptive fields in biological vision (Lindeberg 1997, 2001, 2011; Young et al. 2001; Young RA, Lesperance 2001) and for computing spatio-temporal image features and image descriptors for spatio-temporal recognition in computer vision (Zelnik-Manor and Irani 2001; Laptev and Lindeberg 2003, 2004a, b; Laptev et al. 2007; Willems et al. 2008).

**Fig. 12:**
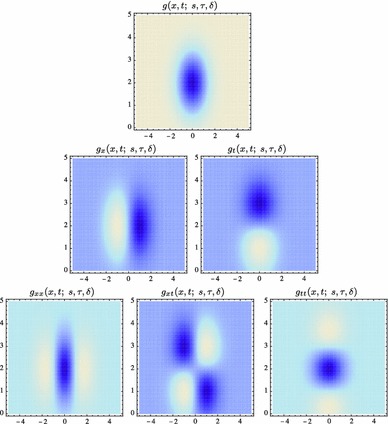
*Space–time separable kernels* $g_{x^{\alpha }t^{\gamma }}(x, t;\; s, \tau , \delta )$ up to order two obtained from the *Gaussian spatio-temporal scale-space* in the case of a 1+1-D space–time ($s = 1$, $\tau = 1$, $\delta = 2$) (*horizontal axis*: space $x$, *vertical axis*: time $t$)

**Fig. 13:**
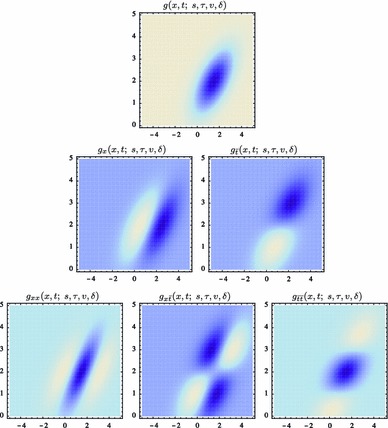
*Velocity-adapted spatio-temporal kernels* $g_{\bar{x}^{\alpha }\bar{t}^{\gamma }}(x, t;\; s, \tau , v, \delta )$ up to order two obtained from the *Gaussian spatio-temporal scale space* in the case of a 1+1D space–time ($s = 1$, $\tau = 1$, $v = 0.75$, $\delta = 2$) (*horizontal axis*: space $x$, *vertical axis*: time $t$)

*Transformation property under Galilean transformations* Under a Galilean transformation of space–time (27), in matrix form written

83 $$\begin{aligned} p' = G_v \, p \end{aligned}$$

corresponding to

84 $$\begin{aligned} \left( \begin{array}{c} x_1' \\ x_2' \\ t' \end{array} \right) = \left( \begin{array}{c@{\quad }c@{\quad }c} 1 &{} 0 &{} v_1 \\ 0 &{} 1 &{} v_2 \\ 0 &{} 0 &{} 1 \\ \end{array} \right) \left( \begin{array}{c} x_1 \\ x_2 \\ t \end{array} \right) , \end{aligned}$$

the corresponding Gaussian spatio-temporal representations are related in an algebraically similar way (64)–(66) as the affine Gaussian scale space with the affine transformation matrix $A$ replaced by a Galilean transformation matrix $G_v$. In other words, if two spatio-temporal image patterns $f_L$ and $f_R$ are related by a Galilean transformation encompassing a translation $\Delta p = (\Delta x_1, \Delta x_2, \Delta t)^T$ in space–time

85 $$\begin{aligned} f_L(\xi ) = f_R(\eta ) \quad \text{ where } \quad \eta = G_v \, \xi + \Delta p \end{aligned}$$

and if corresponding spatio-temporal scale-space representations are defined according to

86 $$\begin{aligned} L(\cdot ;\; \varSigma _L)&= g(\cdot ;\; \varSigma _L) * f_L(\cdot ) \end{aligned}$$

87 $$\begin{aligned} R(\cdot ;\; \varSigma _R)&= g(\cdot ;\; \varSigma _R) * f_R(\cdot ) \end{aligned}$$

for general spatio-temporal covariance matrices $\varSigma _L$ and $\varSigma _R$ of the form (77), then these spatio-temporal scale-space representations will be related according to

88 $$\begin{aligned} L(x;\; \varSigma _L) = R(y;\; \varSigma _R) \end{aligned}$$

where

89 $$\begin{aligned} \varSigma _R = G_v \, \varSigma _L \, G_v^T \end{aligned}$$

and

90 $$\begin{aligned} y = G_v \, x + \Delta p. \end{aligned}$$

### Time-causal spatio-temporal receptive fields

If we on the other hand with regard to real-time biological vision want to respect both temporal causality and temporal recursivity, we obtain different families of receptive fields. Specifically, two different families of time-causal receptive fields can be derived depending on whether we require (i) a continuous semigroup structure over a continuum of temporal scales or (ii) fixate the temporal scale levels to be discrete a priori.

*Time-causal semigroup* Given the requirements of (i) *linearity* and (ii) spatial and temporal *shift invariance*, we require the scale-space kernels to be (iii) *time-causal* and require the visual front end to be (iv) *time recursive* in the sense that the internal image representations $L(x, t;\; s, \tau )$ at different spatial scales $s$ and temporal scales $\tau$ do also constitute a sufficient internal temporal memory $M(x, t)$ of the past, without any further need for temporal buffering. To adapt the convolution semigroup structure to a time-recursive setting, we require the spatio-temporal scale-space concept

91 $$\begin{aligned} L(\cdot , t;\; s, \cdot ) = \mathcal{T}_{s, t} \, L(\cdot , 0;\; 0, \cdot ) \end{aligned}$$

to be generated by a (v) *two-parameter semigroup* over spatial scales $s$ and time $t$

92 $$\begin{aligned} \mathcal{T}_{s_1, t_1} \, \mathcal{T}_{s_2, t_2} = \mathcal{T}_{s_1+s_2, t_1+t_2}. \end{aligned}$$

Then, it can be shown (Lindeberg 2011, Theorem 17, page 78) that provided we impose (vi) certain *regularity properties* on the semigroup in terms of Sobolev norms to ensure differentiability (Lindeberg 2011, Appendix E), then (vii) the *time-recursive formulation of non-enhancement of local extrema* in Eq. (34) with respect to a continuum of both spatial and temporal scale levels implies that the semigroup must satisfy the following system of diffusion equations

93 $$\begin{aligned} \partial _s L&= \frac{1}{2} \nabla _x^T (\varSigma \nabla _x L), \end{aligned}$$

94 $$\begin{aligned} \partial _t L&= - v^T \nabla _x L + \frac{1}{2} \partial _{\tau \tau } L. \end{aligned}$$

In terms of receptive fields, this spatio-temporal scale space can be computed by convolution kernels of the form

95 $$\begin{aligned} h(x, t;\; s, \tau ;\; \varSigma , v)&= g(x - v t;\; s;\; \varSigma ) \, \phi (t;\; \tau ) \nonumber \\&= \frac{1}{2 \pi s \sqrt{\det \varSigma }} \, e^{-(x - v t)^T \varSigma ^{-1} (x - v t)/2s} \, \nonumber \\&\quad \times \frac{1}{\sqrt{2 \pi } \, t^{3/2}} \, \tau \, e^{-\tau ^2/2t} \end{aligned}$$

where

- $g(x - v t;\; s;\; \varSigma )$ is a *velocity-adapted 2D affine Gaussian kernel* with spatial covariance matrix $\varSigma$ and
- $\phi (t;\; \tau )$ is a *time-causal smoothing kernel over time* with temporal scale parameter $\tau$, which is related to the regular one-dimensional Gaussian kernel according to $\phi (t;\; \tau ) = - \partial _{\tau } g(\tau ;\; t)$. (Please note the shift of the order of the arguments between $\phi$ and $g$.)

**Fig. 14:**
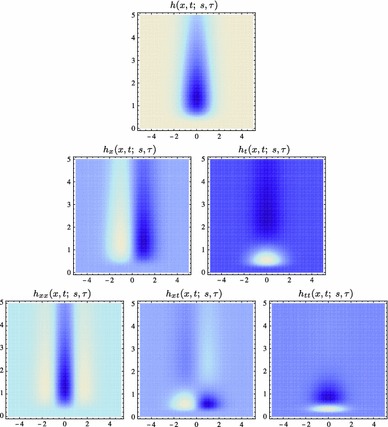
*Space–time separable kernels* $h_{x^{\alpha }t^{\gamma }}(x, t;\; s, \tau , v)$ up to order two obtained from the *time-causal spatio-temporal scale space* in the case of a 1+1D space–time ($s = 1$, $\tau = 2$) (*horizontal axis*: space $x$, *vertical axis*: time $t$)

**Fig. 15:**
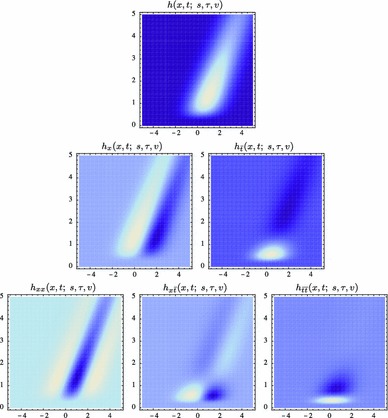
*Velocity-adapted spatio-temporal kernels* $h_{\bar{x}^{\alpha }\bar{t'}^{\gamma }}(x, t;\; s, \tau , v)$ up to order two obtained from the *time-causal spatio-temporal scale space* in the case of a 1+1D space–time ($s = 1$, $\tau = 2$, $v = 0.75$) (*horizontal axis*: space $x$, *vertical axis*: time $t$)

From these kernels, spatio-temporal partial derivatives and velocity-adapted derivatives can be computed in a corresponding manner (81) and (82) as for the Gaussian spatio-temporal scale-space concept. Figures 14 and 15 show examples of such time-causal spatio-temporal kernels with their partial spatio-temporal derivatives in the space–time separable case with $v = 0$

96 $$\begin{aligned} (\partial _{x^{\alpha } t^{\beta }} h)(x, t;\; s, \tau ;\; \varSigma , 0) \!=\! (\partial _{x^{\alpha }} g)(x;\; s;\; \varSigma ) \, (\partial _{t^{\beta }} \phi )(t;\; \tau )\nonumber \\ \end{aligned}$$

and for the velocity-adapted case with $v \ne 0$

97 $$\begin{aligned}&(\partial _{x^{\alpha } {\bar{t}}^{\beta }} h)(x, t;\; s, \tau ;\; \varSigma , v)\nonumber \\&\quad = (\partial _{x^{\alpha }} g)(x-vt;\; s;\; \varSigma ) \, (\partial _{t^{\beta }} \phi )(t;\; \tau ). \end{aligned}$$

The time-causal smoothing kernel $\phi (t;\; \tau )$ has been previously used for modelling heat conduction in solids by (Carslaw and Jaeger (1959), section 14.2) and also been derived by Fagerström (2005) as one member in a family of self-similar kernels obtained from the assumption of scale invariance.

*Truncated exponential kernels/first-order integrators* If we on the other hand fixate the temporal scale levels to be discrete *a priori*, then an alternative model for time-causal and time-recursive receptive fields can be obtained by performing the temporal smoothing using convolution with *truncated exponential functions*

98 $$\begin{aligned} h_\mathrm{exp}(t;\; \mu _i) = \left\{ \begin{array}{l@{\quad }l} \frac{1}{\mu _i} e^{-t/\mu _i} &{} t \ge 0 \\ 0 &{} t < 0 \end{array} \right. \end{aligned}$$

with the composition of $k$ such kernels

99 $$\begin{aligned} h_\mathrm{composed}(t;\; \mu ) = *_{i=1}^{k} h_\mathrm{exp}(t;\; \mu _i) \end{aligned}$$

having a Laplace transform of the form

100 $$\begin{aligned} H_\mathrm{composed}(q;\; \mu )&= \int \limits _{t = - \infty }^{\infty } (*_{i=1}^{k} h_\mathrm{exp}(t;\; \mu _i)) \, e^{-qt} \, \hbox {d}t \nonumber \\&= \prod _{i=1}^{k} \frac{1}{1 + \mu _i q}, \end{aligned}$$

mean value (temporal delay)

101 $$\begin{aligned} \delta _k = M(h_\mathrm{composed}(\cdot ;\; \mu )) = \sum _{t=1}^{k} \mu _i \end{aligned}$$

and variance (temporal extent)

102 $$\begin{aligned} \tau _k = V(h_\mathrm{composed}(\cdot ;\; \mu )) = \sum _{t=1}^{k} \mu _i^2. \end{aligned}$$

When treated as one-dimensional functions over time only, such temporal smoothing kernels do also obey basic scale-space properties in the sense of guaranteeing non-creation of new local extrema or zero-crossings with increasing scale (Lindeberg 1990; Lindeberg and Fagerström 1996). Moreover, they are inherently time recursive and obey a temporal update rule between adjacent temporal scale levels $t_{k-1}$ and $\tau _k$ of the following form:

103 $$\begin{aligned} \partial _t L(t;\; \tau _k) = \frac{1}{\mu _k} \left( L(t;\; \tau _{k-1}) - L(t;\; \tau _k) \right) . \end{aligned}$$

Such first-order integrators over time can also be used as an idealized computational model for temporal processing in biological neurons [see Fig. 18 for an illustration and also (Koch (1999), Chaps. 11–12) regarding physical modelling of the information transfer in dendrites of neurons].

In the absence of further information, it is natural to distribute the temporal scale levels according to a geometric series, corresponding to a uniform distribution in units of *effective temporal scale* $\tau _\mathrm{eff} = \log \tau$:

104 $$\begin{aligned} \tau _k = \gamma ^{k-1} \, \tau _{\min } \qquad \text{ where } \qquad \gamma = \left( \frac{\tau _{\max }}{\tau _{\min }} \right) ^{\frac{1}{K-1}} \end{aligned}$$

for $k = 1 \ldots K$ which by the additive property of variances between adjacent scales

105 $$\begin{aligned} \tau _{k+1} = \tau _{k} + \mu _k^2 \end{aligned}$$

implies that the time constants of the individual temporal smoothing stages should be chosen according to

106 $$\begin{aligned} \mu _k = \sqrt{\tau _{\min } \, (\gamma - 1)} \, \gamma ^{(k-1)/2}. \end{aligned}$$

**Fig. 16:**
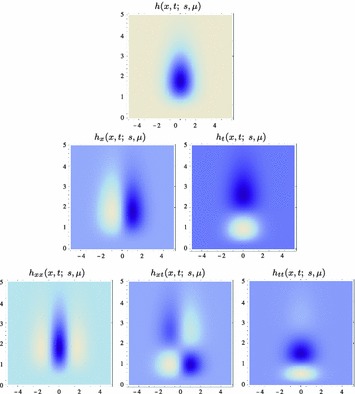
*Space–time separable kernels* $g_{x^{\alpha }t^{\gamma }}(x, t;\; s, \tau )$ up to order two corresponding to the combination of a cascade of $k=7$ time-causal and time-recursive first-order integrators over the temporal domain with a Gaussian scale space over the spatial domain in the case of a 1+1D space–time ($s = 1$, $\tau = 1$) and using a self-similar distribution of the scale levels according to Eqs. (104) and (106) (*horizontal axis*: space $x$, *vertical axis*: time $t$)

**Fig. 17:**
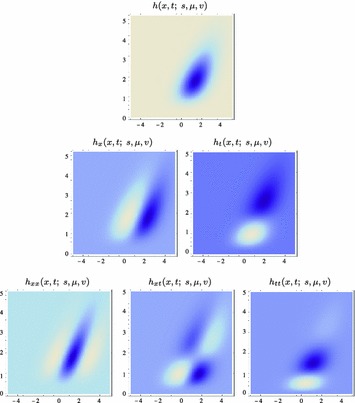
*Velocity-adapted spatio-temporal kernels* $g_{\bar{x}^{\alpha }\bar{t}^{\gamma }}(x, t;\; s, \tau , v)$ up to order two obtained by combining a cascade of $k=7$ time-causal and time-recursive first-order integrators over the temporal domain with a Gaussian scale space over the spatial domain in the case of a 1+1D space–time ($s = 1$, $\tau = 1$, $v = 0.75$) and using a self-similar distribution of the scale levels according to Eqs. (104) and (106) (*horizontal axis*: space $x$, *vertical axis*: time $t$)

If we combine these purely temporal smoothing kernels with the general form of spatio-temporal kernels

107 $$\begin{aligned}&T_\mathrm{space-time}(x, t;\; s, \tau ;\; \varSigma , v)\nonumber \\&\quad = g(x - v t;\; s;\; \varSigma ) \, T_\mathrm{time}(t;\; \tau ) \end{aligned}$$

as obtained from a principled axiomatic treatment over the joint space–time domain for the two other spatio-temporal scale-space concepts according to Eqs. (79) and (95), we obtain an additional class of time-causal and time-recursive spatio-temporal receptive fields with the complementary restriction that the temporal scale parameter has to be discretized already in the theory and that temporal covariance cannot hold exactly for temporal scale levels that have been determined beforehand (see Figs. 16 and 17 for illustrations in the case of a $1+1D$ space–time). In contrast to the time-causal smoothing kernel $\phi (t;\; \tau )$, these kernels do therefore not allow for a continuous semigroup structure over temporal scales.

**Fig. 18:**
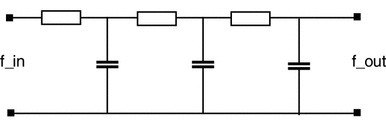
Electric wiring diagram consisting of a set of resistors and capacitors that emulate a series of first-order integrators coupled in cascade, if we regard the time-varying voltage $f_\mathrm{in}$ as representing the time-varying input signal and the resulting output voltage $f_\mathrm{out}$ as representing the time-varying output signal at a coarser temporal scale. According to the theory of temporal scale-space kernels for one-dimensional signals (Lindeberg 1990; Lindeberg and Fagerström 1996), the corresponding equivalent truncated exponential kernels are the only primitive temporal smoothing kernels that guarantee both temporal causality and non-creation of local extrema (alternatively zero-crossings) with increasing temporal scale

### Distributions of spatio-temporal receptive fields

**Fig. 19:**
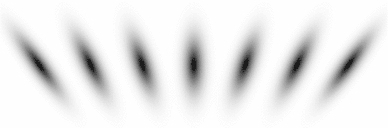
Spatio-temporal receptive fields corresponding to a self-similar distribution of velocity values $v$ for a $1+1D$ space–time for a fixed spatial scale $s$ and a fixed temporal scale $\tau$. In the most idealized version of the theory, one can think of spatio-temporal receptive fields corresponding to all velocity values $v$ being present at any image position $x$. When implementing this receptive field model using a limited number of receptive fields, an additional issue arises of how to distribute the receptive fields over the spatial positions $x$ and the filter parameters $s$, $\tau$, and $v$ (*horizontal dimension*: space $x$, *vertical dimension*: time $t$)

**Fig. 20:**
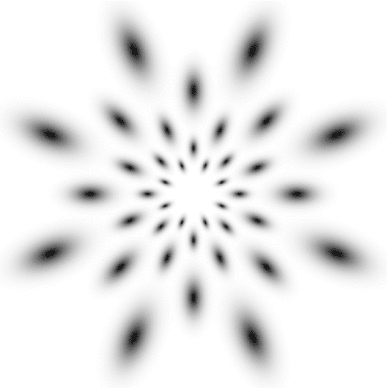
Spatio-temporal receptive fields corresponding to a uniform distribution of motion directions and a self-similar distribution over spatial scales $s$ for a $2+1D$ space–time with the temporal dimension suppressed. In the most idealized version of the theory, one can think of spatio-temporal receptive fields corresponding to all velocity vectors $v$, spatial scales $s$, and temporal scales $\tau$ as being present at any image position $x = (x_1, x_2)^T$. If the spatial components of these receptive fields are additionally allowed to have different spatial shapes, the variability over image velocities should also be extended with a variability over spatial covariance matrices $\varSigma$. When implementing this receptive field model using a limited number of receptive fields, an additional issue arises of how to distribute the receptive fields over the spatial positions $x$ and the filter parameters $s$, $\tau$, $v$, and $\varSigma$ (*horizontal dimension*: spatial coordinate $x_1$, *vertical dimension*: spatial coordinate $x_2$)

Figures 19 and 20 show distributions of velocity-adapted receptive fields over image velocities, in Fig. 19 for a $1+1D$ space–time showing both the spatial and the temporal dimensions and in Fig. 20 for a $2+1D$ space–time showing only the spatial dimensions.

### Geometric covariance properties

The time-causal spatio-temporal scale-space concept given by (95) is *closed* under (i) *rescalings* of the spatial and temporal dimensions, (ii) *Galilean transformations* of space–time, and (iii) *affine transformations* in the spatial domain. Hence, it satisfies the natural transformation properties that allow it to handle:

- image data acquired with different spatial and/or temporal *sampling rates*,
- image structures of different spatial and/or temporal *extent*,
- objects at different *distances* from the camera,
- the linear component of *relative motions* between objects in the world and the observer, and
- the linear component of *perspective deformations*.

Similar covariance properties hold also for the Gaussian spatio-temporal scale space. The covariance properties of the time-causal scale-space based on first-order integrators coupled in cascade are somewhat weaker over the temporal domain because of the restriction to discrete temporal scale levels.

## Computational modelling of biological receptive fields

In two comprehensive reviews, DeAngelis et al. (1995), DeAngelis and Anzai (2004) present overviews of spatial and temporal response properties of (classical) receptive fields in the central visual pathways. Specifically, the authors point out the limitations of defining receptive fields in the spatial domain only and emphasize the need to characterize receptive fields in the *joint* space–time domain, to describe how a neuron processes the visual image. Conway and Livingstone (2006) show the result of a corresponding investigation concerning color receptive fields.

In the following, we will describe how the above-mentioned spatial and spatio-temporal scale-space concepts can be used for modelling the spatial, spatio-chromatic, and spatio-temporal response properties of biological receptive fields. Indeed, it will be shown that the Gaussian and time-causal scale-space concepts lead to predictions of receptive field profiles that are qualitatively very similar to *all* the receptive field types presented in DeAngelis et al. (1995), DeAngelis and Anzai (2004), and schematic simplifications of most of the receptive fields shown in Conway and Livingstone (2006).

### LGN neurons

In the lateral geniculate nucleus (LGN), most neurons (DeAngelis et al. 1995; DeAngelis and Anzai 2004)

**Fig. 21:**
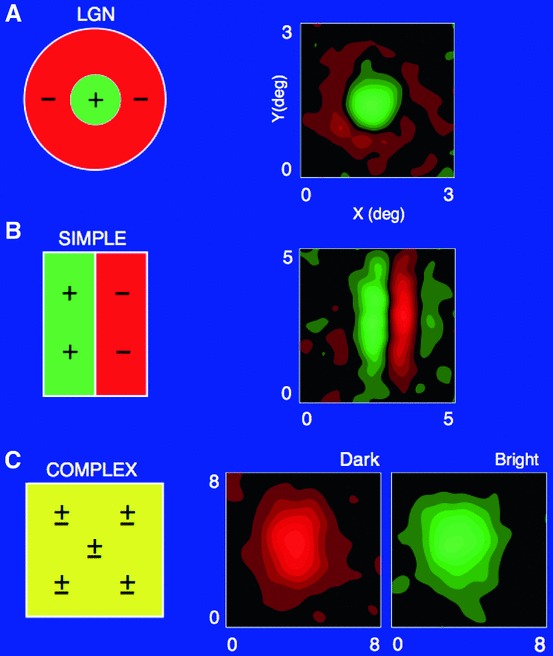
Examples of receptive field profiles in the spatial domain as reported by DeAngelis et al. (1995), DeAngelis and Anzai (2004). **a** Receptive fields in the LGN have approximately circular center-surround responses in the spatial domain. In terms of Gaussian derivatives, this spatial response profile can be modelled by the Laplacian of the Gaussian $\nabla ^2 g(x;\; t)$ (see Fig. 22a). **b** Simple cells in the cerebral cortex do usually have strong directional preference in the spatial domain. In terms of Gaussian derivatives, this spatial response can be modelled as a directional derivative of an elongated affine Gaussian kernel (see Fig. 22b). **c** Complex cells are nonlinear and do not obey the superposition principle

**Fig. 22:**
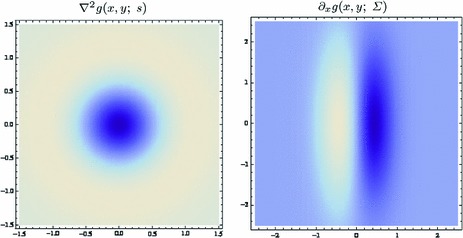
Idealized models of receptive fields over the spatial domain: (*left*) The Laplacian of an isotropic two-dimensional Gaussian smoothing kernel over a spatial domain $\nabla ^2 g(x, y;\; s) = (x^2 + y^2 - 2s)/(2 \pi s^3) \exp (-(x^2+y^2)/2s)$ (here with $s = 0.4$) can be used as a model for the circular center-surround responses in the LGN illustrated in Fig. 21a. More generally, this Laplacian of Gaussian with a rather wide range of scales can be used as a model for retinal or LGN receptive fields of wide size ranges, depending on the scale level and the distance from the fovea (see also Sect. 7). (*right*) First-order directional derivatives of anisotropic affine Gaussian kernels (here aligned to the coordinate directions $\partial _x g(x, y;\; \varSigma ) = \partial _x g(x, y;\; \lambda _x, \lambda _y) = - \frac{x}{\lambda _x} 1/(2 \pi \sqrt{\lambda _x \lambda _y}) \exp (-x^2/2 m\lambda _x -y^2/2\lambda _y)$ and with $\lambda _x = 0.2$ and $\lambda _y = 2$) can be used as a model for simple cells with a strong directional preference as illustrated in Fig. 21b. More generally, elongated receptive fields can also have different degrees of elongation as described in Sect. 6.3.1 and illustrated in Fig. 8

- have approximately *circular center-surround* organization in the spatial domain (see Fig. 21a) and
- most of the receptive fields are *separable in space–time* (Fig. 22).

**Fig. 23:**
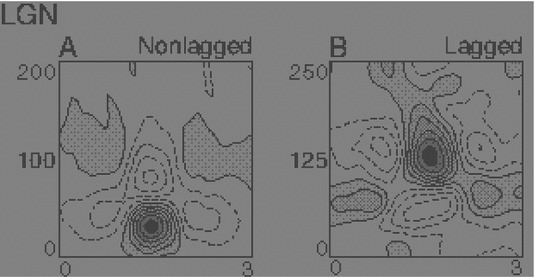
Examples of *space–time separable receptive field profiles in the LGN* as reported by DeAngelis et al. (1995), DeAngelis and Anzai (2004). There are two main categories of such cells; **a** for a non-lagged cell, the first temporal lobe dominates, while **b** for a lagged cell the second temporal lobe is strongest. In terms of the spatio-temporal receptive field model presented in this paper, non-lagged cells can be modelled by first-order temporal derivatives, while the shape of lagged cells resembles second-order temporal derivatives (see Fig. 24) (*horizontal dimension*: space $x$, *vertical dimension*: time $t$)

There are two main classes of temporal responses for such cells:

- a “non-lagged cell” is defined as a cell for which the first temporal lobe is the largest one (Fig. 23a), whereas
- a “lagged cell” is defined as a cell for which the second lobe dominates (Fig. 23b).

Such temporal response properties are typical for *first- and second-order temporal derivatives* of a time-causal temporal scale-space representation. For the first-order temporal derivative of a time-causal temporal scale-space kernel, the first peak is strongest, whereas the second peak is the most dominant one for second-order temporal derivatives. The spatial response, on the other hand, shows a high similarity to a *Laplacian of a Gaussian*.

Within the above-mentioned spatio-temporal scale-space theory, we can approximate the qualitative shape of these circular center-surround receptive fields in the LGN with the following idealized model:

108 $$\begin{aligned}&h_\mathrm{LGN}(x_1, x_2, t;\; s, \tau )\nonumber \\&\quad = \pm (\partial _{x_1x_1} + \partial _{x_2x_2}) \, g(x_1, x_2;\; s) \, \partial _{{t'}^n} \, h(t;\; \tau ) \end{aligned}$$

where

- $\pm$ determines the polarity (on-center/off-surround versus off-center/on-surround),
- $\partial _{x_1 x_1} + \partial _{x_2 x_2}$ denotes the spatial Laplacian operator,
- $g(x_1, x_2;\; s)$ denotes a rotationally symmetric spatial Gaussian,
- $\partial _{{t'}}$ denotes a temporal derivative operator with respect to a possibly self-similar transformation of time $t' = t^{\alpha }$ or $t' = \log t$ such that $\partial _{{t'}} = t^{\kappa } \, \partial _t$ for some constant $\kappa \in [0, 1]$ ( Lindeberg 2011, Sect. 5.1, pages 59–61)^13^,
- $h(t;\; \tau )$ is a temporal smoothing kernel over time corresponding to the time-causal smoothing kernel $\phi (t;\; \tau ) = \tfrac{1}{\sqrt{2 \pi } \, t^{3/2}} \, \tau \, e^{-\tau ^2/2t}$ in (95), a non-causal time-shifted Gaussian kernel $g(t;\; \tau , \delta ) = \tfrac{1}{\sqrt{2 \pi \tau }} e^{-(t - \delta )^2/2 \tau }$ according to (76) or a time-causal kernel corresponding to a set of first-order integrators over time coupled in cascade having a Laplace transform $H_\mathrm{composed}(q;\; \mu ) = \prod _{i=1}^{k} \frac{1}{1 + \mu _i q}$ according to (99),
- $n$ is the order of temporal differentiation,
- $s$ is the spatial scale parameter and
- $\tau$ is the temporal scale parameter.

Figure 22a shows an illustration of the spatial response properties of such a receptive field. This model can also be used for modelling on-center/off-surround and off-center/on-surround receptive fields in the retina.

Regarding the spatial domain, the model in terms of spatial Laplacians of Gaussians $(\partial _{x_1 x_1} + \partial _{x_2 x_2 }) \, g(x_1, x_2;\; s)$ is closely related to differences in Gaussians, which have previously been shown to constitute a good approximation of the spatial variation of receptive fields in the retina and the LGN (Rodieck 1965). This property follows from the fact that the rotationally symmetric Gaussian satisfies the isotropic diffusion equation

109 $$\begin{aligned} \frac{1}{2} \nabla ^2 L(x;\; t)&= \partial _t L(x;\; t) \approx \frac{L(x;\; t + \Delta t) - L(x;\; t)}{\Delta t}\nonumber \\&= \frac{DOG(x;\; t, \Delta t)}{\Delta t} \end{aligned}$$

which implies that differences in Gaussians can be interpreted as approximations of derivatives over scale and hence to Laplacian responses. Conceptually, this implies very good agreement with the spatial component of the LGN model (108) in terms of Laplacians of Gaussians. More recently, Bonin et al. (2005) have found that LGN responses in cats are well described by difference in Gaussians and temporal smoothing complemented by a nonlinear contrast gain control mechanism (not modelled here).

Concerning the application of the Laplacian of Gaussian model for on-center/off-surround and off-center/on-surround receptive fields in the retina, it should be emphasized that the retina also contains other types of receptive fields that are not modelled here, such as brisk transient (Y) ganglion cells that respond to rapid transients and directional selective ganglion cells that respond to visual motion (Wässle 2004).

**Fig. 24:**
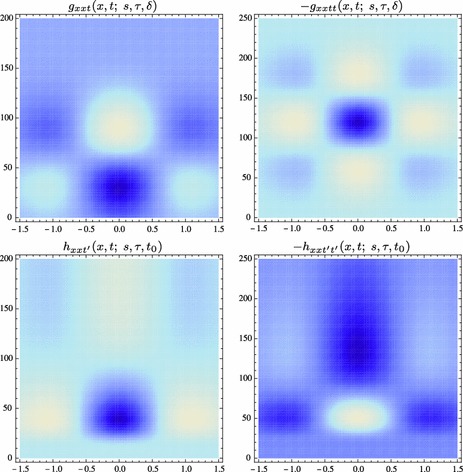
Idealized models of space–time separable receptive fields as obtained from the spatio-temporal scale-space concepts with $v = 0$: (*upper left*) Gaussian spatio-temporal kernel $g_{xxt}(x, t;\; s, \tau , \delta ) = g_{xx}(x;\; s) \, g_t(t;\; \tau , \delta )$ with $s = 0.4, \tau = 30^2, \delta = 60$. (*upper right*) Gaussian spatio-temporal kernel $g_{xxtt}(x, t;\; s, \tau , \delta ) = g_{xx}(x;\; s) \, g_{tt} (t;\; \tau , \delta )$ with $s = 0.3, \tau = 35^2, \delta = 120$. (*lower left*) Time-causal spatio-temporal kernel $h_{xxt'} h(x, t;\; s, \tau ) = g_{xx}(x;\; s) \, \phi _{t'}(t;\; \tau , \delta )$ with $s = 0.4, \tau = 17$. (*lower right*) Time-causal spatio-temporal kernel $h_{xxt't'} h(x, t;\; s, \tau ) = g_{xx}(x;\; s) \, \phi _{t't'}(t;\; \tau , \delta )$ with $s = 0.4, \tau = 25$. For the time-causal kernels, the temporal derivatives have been computed using the transformed temporal derivative operator $\partial _{t'} \sim t^{\kappa } \, \partial _t$, here with $\kappa = 1/2$. Compare the qualitative shapes of these kernels with the kernels in with Fig. 23 (*horizontal dimension*: space $x$, *vertical dimension*: time $t$)

*Note:* In all illustrations in Sect. 6, where spatial and spatio-temporal derivative expressions are aligned to biological data, the unit for the spatial scale parameter $s$ corresponds to $[\text{ degrees }^2]$ of visual angle and the units for the temporal scale parameter $\tau$ in the Gaussian spatio-temporal scale-space representation are $[\text{ milliseconds }^2]$, whereas the units for the temporal scale parameter $\tau$ in the time-causal spatio-temporal scale-space representation are $[\sqrt{\text{ milliseconds }}]$. For image velocities $v$ of velocity-adapted filters, the units are $[\text{ degrees/millisecond }]$. The reason why the units are different for the three types of spatio-temporal scale spaces is that the dimensionality of the temporal scale parameter is different in each of these spatio-temporal scale-space concepts.

Figure 24 shows the spatio-temporal response properties of space–time separable receptive field over a 1+1D spatio-temporal domain according to the model in Eq. (108) for a first-order temporal derivative in combination with a second-order spatial derivative in the left column and a second-order temporal derivative in combination with a second-order spatial derivative in the right column. These kernels were chosen to mimic the qualitative behaviour of the biological receptive fields shown in Fig. 23.

### Double-opponent spatio-chromatic cells

**Fig. 25:**
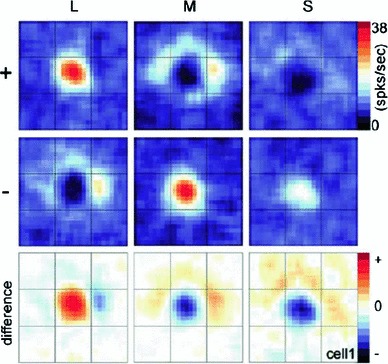
Spatio-chromatic receptive field response of a *double-opponent neuron* as reported by (Conway and Livingstone (2006), Fig. 2, page 10831) with the color channels *L*, *M* and *S* essentially corresponding to red, green, and blue, respectively (from these *L*, *M*, and *S* color channels, corresponding red/green and yellow/blue color-opponent channels can be formed from the differences between *L* to *M* and between $L+M$ to *S*)

In a study of spatio-chromatic response properties of V1 neurons in the alert macaque monkey, Conway and Livingstone (2006) describe receptive fields with approximately circular red/green and yellow/blue color-opponent response properties over the spatio-chromatic domain, see Fig. 25. Such cells are referred to as *double-opponent cells*, since they simultaneously compute both spatial and chromatic opponency. According to Conway and Livingstone (2006), this cell type can be regarded as the first layer of spatially opponent color computations.

**Fig. 26:**
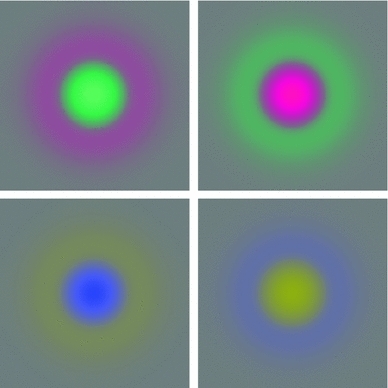
Idealized models of spatio-chromatic receptive fields over the spatial domain corresponding to the application of the Laplacian operator to positive and negative *red*/*green* and *yellow*/*blue* color-opponent channels, respectively

If we, motivated by the previous application of Laplacian of Gaussian functions to model rotationally symmetric on-center/off-surround and off-center/on-surround receptive fields in the LGN (108), apply the Laplacian of the Gaussian operator to red/green and yellow/blue color-opponent channels, respectively, we obtain equivalent spatio-chromatic receptive fields corresponding to red-center/green-surround, green-center/red-surround, yellow-center/blue-surround, or blue-center/yellow-surround, respectively, as shown in Fig. 26 and corresponding to the following spatial receptive field model applied to the RGB channels

110 $$\begin{aligned}&h_\mathrm{double-opponent}(x_1, x_2;\; s)\nonumber \\&\quad = \pm (\partial _{x_1x_1} + \partial _{x_2x_2}) \, g(x_1, x_2;\; s) \left( \begin{array}{c@{\quad }c@{\quad }c} \tfrac{1}{2} &{} - \tfrac{1}{2} &{} 0 \\ \tfrac{1}{2} &{} \tfrac{1}{2} &{} -1 \\ \end{array} \right) . \end{aligned}$$

Hence, these spatio-chromatic receptive fields can be used as an idealized model for the spatio-chromatic response properties for double-opponent cells.

### Simple cells

In V1, the receptive fields are generally different from the receptive fields in the LGN in the sense that they are (DeAngelis et al. 1995; DeAngelis and Anzai 2004):

- *oriented in the spatial domain* and
- *sensitive to specific stimulus velocities.*

Cells (i) for which there are precisely localized “on” and “off” subregions with (ii) spatial summation within each subregion, (iii) spatial antagonism between on- and off-subregions, and (iv) whose visual responses to stationary or moving spots can be predicted from the spatial subregions are referred to as *simple cells* (Hubel and Wiesel 1959, 1962).

#### Spatial dependencies

We can express an idealized scale-space model for the *spatial component* of this orientation dependency according to

111 $$\begin{aligned} h_\mathrm{space}(x_1, x_2;\; s) = (\cos \varphi \, \partial _{x_1} + \sin \varphi \, \partial _{x_2})^m \, g(x_1, x_2;\; \varSigma )\nonumber \\ \end{aligned}$$

where

- $\partial _{\varphi } = \cos \varphi \, \partial _{x_1} + \sin \varphi \, \partial _{x_2}$ is a directional derivative operator,
- $m$ is the order of spatial differentiation, and
- $g(x_1, x_2;\; \varSigma )$ is an affine Gaussian kernel with spatial covariance matrix $\varSigma$ as can be parameterized according to (68)

where the direction $\varphi$ of the directional derivative operator should preferably be aligned to the orientation $\theta$ of one of the eigenvectors of $\varSigma$.

In the specific case when the covariance matrix is proportional to a unit matrix $\varSigma = s \, I$, with $s$ denoting the spatial scale parameter, these directional derivatives correspond to regular Gaussian derivatives as proposed as a model for spatial receptive fields by Koenderink and Doorn (1987, 1992). The use of non-isotropic covariance matrices does on the other hand allow for a higher degree of orientation selectivity and does additionally allow for closedness under affine transformations (affine covariance).

This idealized model can also be extended to recurrent intracortical feedback mechanisms as formulated by Somers et al. (1995) and Sompolinsky and Shapley (1997) by starting from the equivalent formulation in terms of the non-isotropic diffusion equation

112 $$\begin{aligned} \partial _s L = \frac{1}{2} \nabla _x^T \left( \varSigma _0 \nabla _x L \right) \end{aligned}$$

with the covariance matrix $\varSigma _0$ locally adapted^14^ to the statistics of image data in a neighborhood of each image point; see Weickert (1998) and Almansa and Lindeberg (2000) for the applications of this idea for enhancing local directional image structures in computer vision.

*Relations to Gabor functions* Based on the work by Marcelja (1980), Gabor functions

113 $$\begin{aligned} G(x;\; s, \omega ) = e^{-i\omega x} \, g(x;\; s) \end{aligned}$$

have been frequently used for modelling spatial receptive fields (Jones and Palmer 1987a, b; Ringach 2002) motivated by their property of minimizing the uncertainty relation. This motivation can, however, be questioned on both theoretical and empirical grounds. Stork and Wilson (1990) argue that (i) only complex-valued Gabor functions that cannot describe single receptive field minimize the uncertainty relation, (ii) the real functions that minimize this relation are Gaussian derivatives rather than Gabor functions, and (iii) comparisons among Gabor and alternative fits to both psychophysical and physiological data have shown that in many cases, other functions (including Gaussian derivatives) provide better fits than Gabor functions do.

Conceptually, the ripples of the Gabor functions, which are given by complex sine waves, are related to the ripples of Gaussian derivatives, which are given by Hermite functions. A Gabor function, however, requires the specification of a scale parameter and a spatial frequency, whereas a Gaussian derivative requires a scale parameter and the order of differentiation (per spatial dimension). With the Gaussian derivative model, receptive fields of different orders can be mutually related by derivative operations and be computed from each other by nearest-neighbor operations. The zero-order receptive fields as well as the derivative-based receptive fields can be modelled by diffusion equations and can therefore be implemented by computations between neighboring computational units.

In relation to invariance properties, the family of affine Gaussian kernels is closed under affine image deformations, whereas the family of Gabor functions obtained by multiplying rotationally symmetric Gaussians with sine and cosine waves is not closed under affine image deformations. This means that it is not possible to compute truly affine invariant image representations from such Gabor functions. Instead, given a pair of images that are related by a non-uniform image deformation, the lack of affine covariance implies that there will be a systematic bias in the image representations derived from such Gabor functions, corresponding to the difference between the backprojected Gabor functions in the two image domains. If using receptive profiles defined from directional derivatives of affine Gaussian kernels, it will on the other hand be possible to compute provably affine invariant image representations.

With regard to invariance to multiplicative illumination variations, the even cosine component of a Gabor function does in general not have its integral equal to zero, which means that the illumination invariant properties under multiplicative illumination variations or exposure control mechanisms described in Sect. 2.3 do not hold for Gabor functions.

In this respect, the Gaussian derivative model is simpler, it can be related to image measurements by differential geometry, be derived axiomatically from symmetry principles, be computed from a minimal set of connections and allows for provable invariance properties under locally linearized image deformations (affine transformations) as well as local multiplicative illumination variations and exposure control mechanisms. Young (1987) has more generally shown how spatial receptive fields in cats and monkeys can be well modelled by Gaussian derivatives up to order four.

In the area of computer vision, a multi-scale differential geometric framework in terms of Gaussian derivatives and closely related operators has become an accepted and de facto standard for defining image features for feature detection, feature classification, stereo matching, motion estimation, object recognition, spatio-temporal recognition, shape analysis, and image enhancement. Specifically, the formulation of image primitives in terms of scale-space derivatives makes it possible to use tools from differential geometry for deriving relationships between image features and physical properties of objects in the environment, allowing for computationally operational and theoretically well-founded modelling of possibilities or constraints for visual perception.

**Fig. 27:**
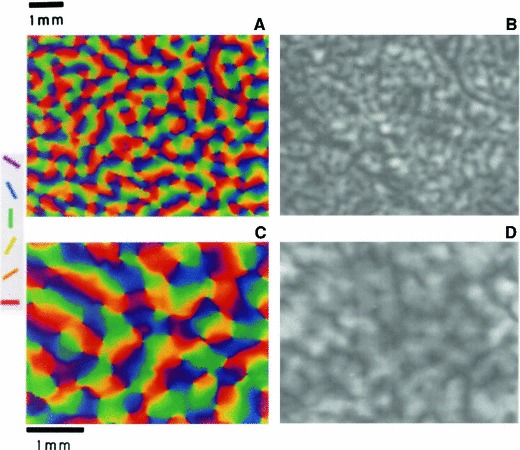
(*left*) Orientation maps from the striate cortex using a color coding of the orientation preference with *red* corresponding to horizontal and *green* to vertical. (*right*) Selective maps with bright values corresponding to high orientation selectivity and *dark values* corresponding to low orientation selectivity (from Blasdel 1992)

*Orientation maps* Optical imaging techniques have shown that orientation selective cells that respond best to one orientation form are grouped together in highly ordered patches and that these iso-orientation patches are organized around “orientation centers” that produce characteristic pinwheel-like patterns (Bonhoeffer and Grinvald 1991). Measurements have also shown that the degree of orientation selectivity varies regularly over the cortex and can be different near versus further away from the center of a pinwheel (Blasdel 1992). Specifically, the orientation selectivity has been reported to be lowest at the positions of the centers of the pinwheels (see Fig. 27).

Given the model (111) of orientation selective receptive fields as depending on a spatial covariance matrix $\varSigma$, this property is in good qualitative agreement with a distribution of receptive fields over a population over covariance matrices with different preferred orientations as determined from the eigenvectors of the covariance matrix and different ratios between the scale parameters along the preferred orientations as determined by the square root of the ratio between the eigenvalues of the covariance matrix. Specifically, the property of the orientation selectivity of being lowest at the positions of the centers of the pinwheels would be compatible with the covariance matrix there being close to alternatively closer to a unit matrix, implying that the orientations of the eigenvectors being sensitive to minor perturbations of the covariance matrix, thus causing the ratio between the eigenvalues being close to alternatively closer to one at the center of the pinwheel.

#### Spatio-temporal dependencies

**Fig. 28:**
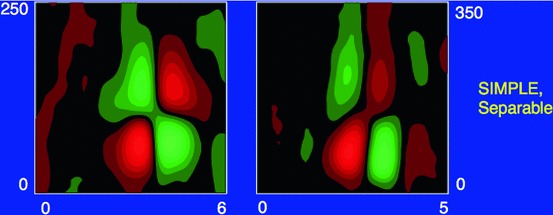
Examples of *space–time separable receptive field profiles in the striate cortex* as reported by DeAngelis et al. (1995), DeAngelis and Anzai (2004): **a** a non-lagged cell reminiscent of a first-order temporal derivative in time and a first-order derivative in space (compare with Fig. 29a) **b** a non-lagged cell reminiscent of a first-order temporal derivative in time and a second-order derivative in space (compare with Fig. 29b) (*horizontal dimension*: space $x$, *vertical dimension*: time $t$)

**Fig. 29:**
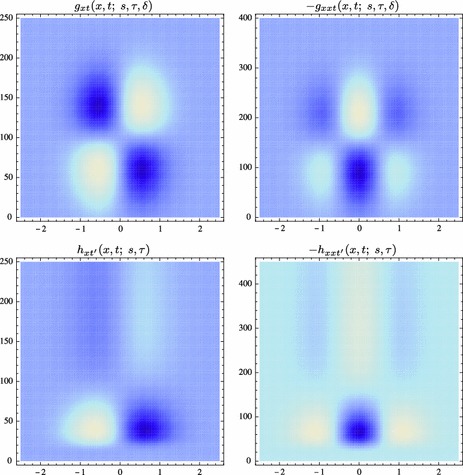
Idealized models of space–time separable receptive fields as obtained from the spatio-temporal scale-space concepts with $v = 0$: (*upper left*) Gaussian spatio-temporal kernel $g_{xt}(x, t;\; s, \tau , \delta ) = g_{x}(x;\; s) \, g_t(t;\; \tau , \delta )$ with $s = 0.3, \tau = 40^2, \delta = 100$. (*upper right*) Gaussian spatio-temporal kernel $g_{xxt}(x, t;\; s, \tau , \delta ) = g_{xx}(x;\; s) g_t(t;\; \tau , \delta )$ with $s = 0.3, \tau = 60^2, \delta = 150$. (*lower left*) Time-causal spatio-temporal kernel $h_{xt'}(x, t;\; s, \tau ) = g_x(x;\; s) \, \phi _{t'}(t;\; \tau , \delta )$ with $s = 0.4, \tau = 17$. (*lower right*) Time-causal spatio-temporal kernel $h_{xxt'}(x, t;\; s, \tau ) = g_{xx}(x;\; s) \, \phi _{t'}(t;\; \tau , \delta )$ with $s = 0.4, \tau = 22$. For the time-causal kernels, the temporal derivatives have been computed using the transformed temporal derivative operator $\partial _{t'} \sim t^{\kappa } \partial _t$, here with $\kappa = 1/2$. Compare the qualitative shapes of these kernels with the kernels in Fig. 28 (*horizontal dimension*: space $x$, *vertical dimension*: time $t$)

**Fig. 30:**
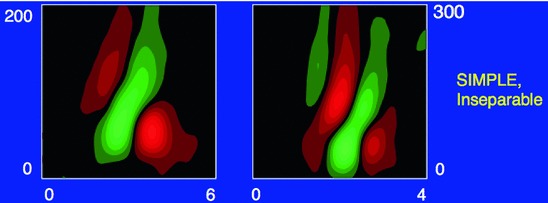
Examples of *non-separable receptive field profiles in the striate cortex* as reported by DeAngelis et al. (1995), DeAngelis and Anzai (2004): **a** a receptive field reminiscent of a second-order derivative in tilted space–time (compare with the left column in Fig. 31) **b** a receptive field reminiscent of a third-order derivative in tilted space–time (compare with the right column in Fig. 31) (*horizontal dimension*: space $x$, *vertical dimension*: time $t$)

**Fig. 31:**
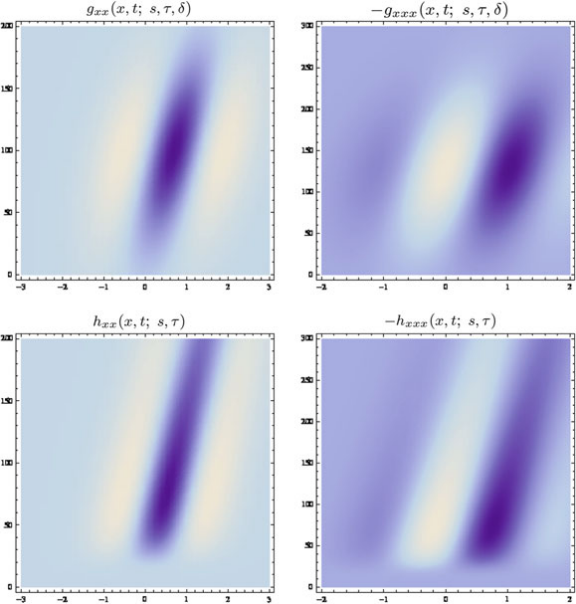
Idealized models of non-separable spatio-temporal receptive obtained by applying velocity-adapted second- and third-order derivative operations in space–time to spatio-temporal smoothing kernels generated by the spatio-temporal scale-space concept. (*middle left*) Gaussian spatio-temporal kernel $g_{xx}(x, t;\; s, \tau , v, \delta )$ with $s = 0.5~\text{ deg }^2, \tau = 50^2~\text{ ms }^2, v = 0.007~\text{ deg/ms }, \delta = 100~\text{ ms }$. (*middle right*) Gaussian spatio-temporal kernel $g_{xxx}(x, t;\; s, \tau , v, \delta )$ with $s = 0.5~\text{ deg }^2, \tau = 60^2~\text{ ms }^2, v = 0.004~\text{ deg/ms }, \delta = 130~\text{ ms }$. (*lower left*) Time-causal spatio-temporal kernel $h_{xx}(x, t;\; s, \tau , v)$ with $s = 0.4~\text{ deg }^2, \tau = 15~\text{ ms }^{1/2}, v = 0.007~\text{ deg/ms }$. (*lower right*) Time-causal spatio-temporal kernel $h_{xxx}(x, t;\; s, \tau , v)$ with $s = 0.4~\text{ deg }^2, \tau = 15~\text{ ms }^{1/2}, v = 0.004~\text{ deg/ms }$ (*horizontal dimension*: space $x$, *vertical dimension*: time $t$). Compare the qualitative shapes of these kernels with the kernels in Fig. 30 (*horizontal dimension*: space $x$, *vertical dimension*: time $t$). To handle objects or events with different relative motions between the object/event and the observer, it is natural to consider families of spatio-temporal receptive fields that are tuned to different image velocities and motion direction in image space, thus leading to a set of velocity-adapted fields tuned to different motion directions and image velocities at every image point (see Figs. 19 and 20 for schematic illustrations)

In the *joint space–time domain*, the spatio-temporal response properties of receptive fields in the striate cortex range from separable (Fig. 28) to strongly inseparable (Fig. 30), where a majority exhibit *marked space–time inseparability.* The temporal profile is reported to be typically biphasic, although some cells are reported to have monophasic or triphasic responses (DeAngelis et al. 1995; DeAngelis and Anzai 2004) (Fig. 29, 31).

In terms of temporal derivatives, a biphasic behavior arises from first-order derivatives, a monophasic behavior from zero-order derivatives, and a triphasic behavior from second-order derivatives. Concerning the oriented spatial response characteristics, there is a high similarity with directional derivatives of Gaussian kernels (Young 1987).

We can state scale-space models of simple cells in V1 with similar properties using either:

- *non-causal Gaussian spatio-temporal derivative kernels*
  114 $$\begin{aligned}&h_\mathrm{Gaussian}(x_1, x_2, t;\; s, \tau , v, \delta ) \nonumber \\&\quad = \partial _{\varphi }^{m_1} \, \partial _{\bot \varphi }^{m_2} \, \partial _{\bar{t}^n} g(x_1, x_2, t;\; s, \tau , v, \delta ) \end{aligned}$$
- *time-causal spatio-temporal derivative kernels*
  115 $$\begin{aligned}&h_\mathrm{time-causal}(x_1, x_2, t;\; s, \tau , v)\nonumber \\&\quad = (\partial _{\bar{x_1}^{\alpha _1} \bar{x_2}^{\alpha _2}} \partial _{\bar{t}^{\beta }} h)(x_1, x_2, t;\; s, \tau , v) \end{aligned}$$

with the non-causal Gaussian spatio-temporal kernels according to (76), the time-causal spatio-temporal kernels according to (95) alternatively of the form (107) with the temporal smoothing based on a cascade of first-order integrators according to (99), and spatio-temporal derivatives or velocity-adapted derivatives of these spatio-temporal kernels in turn defined according to (81) and (82).

For a general orientation of receptive fields with respect to the spatial coordinate systems, these idealized receptive field models can be jointly described in the form

116 $$\begin{aligned}&h_\mathrm{simple cell}(x_1, x_2, t;\; s, \tau , v, \varSigma ) \nonumber \\&\quad = (\cos \varphi \, \partial _{x_1} + \sin \varphi \, \partial _{x_2})^{\alpha _1} (\sin \varphi \, \partial _{x_1} - \cos \varphi \, \partial _{x_2})^{\alpha _2} \nonumber \\&\qquad \times (v_1 \, \partial _{x_1} + v_2 \, \partial _{x_2} + \partial _t)^n \nonumber \\&\qquad \times g(x_1 - v_1 t, x_2 - v_2 t;\; s \, \varSigma ) \, h(t;\; \tau ) \end{aligned}$$

where

- $\partial _{\varphi } = \cos \varphi \, \partial _{x_1} + \sin \varphi \, \partial _{x_2}$ and $\partial _{\bot \varphi } = \sin \varphi \, \partial _{x_1} - \cos \varphi \, \partial _{x_2}$ denote spatial directional derivative operators according to (69) in two orthogonal directions $\varphi$ and $\bot \varphi$,
- $m_1 \ge 0$ and $m_2 \ge 0$ denote the orders of differentiation in the two orthogonal directions in the spatial domain with the overall spatial order of differentiation $m = m_1 + m_2$,
- $v_1 \, \partial _{x_1} + v_2 \, \partial _{x_2} + \partial _t$ denotes a velocity-adapted temporal derivative operator,
- $v = (v_1, v_2)^T$ denotes the image velocity,
- $n$ denotes the order of temporal differentiation,
- $g(x_1 - v_1 t, x_2 - v_2 t;\; \varSigma )$ denotes a spatial affine Gaussian kernel according to (63) that moves with image velocity $v = (v_1, v_2)^T$ in space–time,
- $\varSigma$ denotes a spatial covariance matrix that can be parameterized by two eigenvalues $\lambda _1$ and $\lambda _2$ as well as a spatial orientation $\theta$ of the form (68),
- $h(t;\; \tau )$ is a temporal smoothing kernel over time corresponding to the time-causal smoothing kernel $\phi (t;\; \tau ) = \tfrac{1}{\sqrt{2 \pi } \, t^{3/2}} \, \tau \, e^{-\tau ^2/2t}$ in (95), a non-causal time-shifted Gaussian kernel $g(t;\; \tau , \delta ) = \tfrac{1}{\sqrt{2 \pi \tau }} e^{-(t - \delta )^2/2 \tau }$ according to (76) or a time-causal kernel corresponding to a set of first-order integrators over time coupled in cascade having a Laplace transform $H_\mathrm{composed}(q;\; \mu ) = \prod _{i=1}^{k} \frac{1}{1 + \mu _i q}$ according to (99),
- $s$ denotes the spatial scale and
- $\tau$ denotes the temporal scale.

Figures 24, 29, and 31 show a few examples of separable and inseparable kernels obtained in this way for a 1+1-dimensional space–time. In fact, using this model, it is possible to generate spatio-temporal receptive fields that are qualitatively similar to *all* the linear receptive field types reported from cell recordings in LGN and V1 by DeAngelis et al. (1995), DeAngelis and Anzai (2004).

Young et al. (2001) and Young RA, Lesperance (2001) have also shown how spatio-temporal receptive fields can be modelled by Gaussian derivatives over a spatio-temporal domain, corresponding to the Gaussian spatio-temporal concept described here, although with a different type of parameterization; see also Lindeberg (1997, 2001) for closely related earlier work. These scale-space models can therefore be regarded as *idealized functional and phenomenological models of receptive fields,* whose actual realization can then be implemented in different ways depending on available hardware or wetware.

*Relations to approaches for learning receptive fields from natural image statistics* Work has also been performed on learning receptive field properties and visual models from the statistics of natural image data (Field 1987; van der Schaaf and van Hateren 1996; Olshausen and Field 1996; Rao and Ballard 1998; Simoncelli and Olshausen 2001; Geisler 2008; Hyvärinen et al. 2009; Lörincz et al. 2012) and been shown to lead to the formation of similar receptive fields as found in biological vision. The proposed theory of receptive fields can be seen as describing basic physical constraints under which a learning-based method for the development of receptive fields will operate and the solutions to which an optimal adaptive system may converge to, if exposed to a sufficiently large and representative set of natural image data. Field (1987) as well as Doi and Lewicki (2005) have described how ”natural images are not random, instead they exhibit statistical regularities” and have used such statistical regularities for constraining the properties of receptive fields. The theory presented in this paper can be seen as a theory at a higher level of abstraction, in terms of basic principles that reflect properties of the environment that in turn determine properties of the image data, without need for explicitly constructing specific statistical models for the image statistics. Specifically, the proposed theory can be used for explaining why the above-mentioned statistical models lead to qualitatively similar types of receptive fields as the idealized receptive fields obtained from our theory.

An interesting observation that can be made from the similarities between the receptive field families derived by necessity from the assumptions and receptive profiles found by cell recordings in biological vision is that receptive fields in the retina, LGN, and V1 of higher mammals are very close to *ideal* in view of the stated structural requirements/symmetry properties. In this sense, biological vision can be seen as having adapted very well to the transformation properties of the surrounding world and the transformations that occur when a three-dimensional world is projected to a two-dimensional image domain.

### Spatio-chrom-temporal receptive fields

**Fig. 32:**
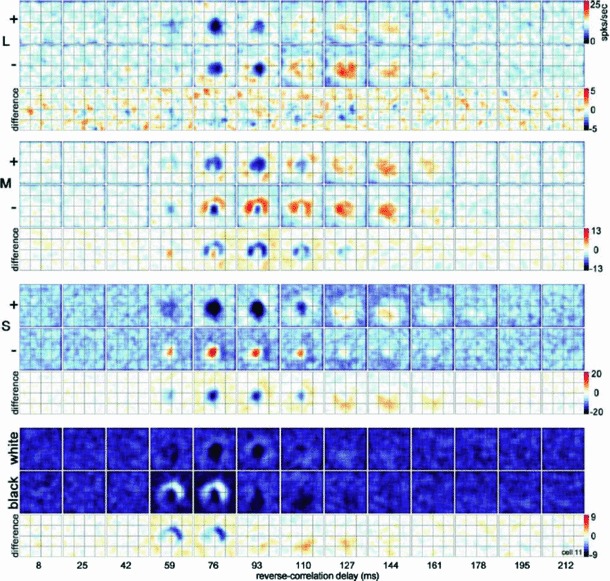
Spatio-temporal response properties of a blue/yellow double-opponent cell as reported by Conway and Livingstone (Conway and Livingstone (2006), Fig. 15, page 10842) with an L+M ON-center and S suppression character, with the color channels *L*, *M*, and *S* essentially corresponding to *red*, *green*, and *blue*, respectively. An idealized model for the spatio-chrom-temporal response properties of this cell can be obtained by combining the spatio-chromatic color-opponent Laplacian receptive fields in Fig. 26 over the spatio-chromatic domain with a space–time separable temporal smoothing filter $h(t;\; \tau )$ over the temporal domain

By extending the spatial derivative operators to spatio-chromatic derivates over color-opponent channels, the color-opponent Laplacian operators in Eq. (110) can in combination with a temporal response function over time be used for modelling the spatio-chrom-temporal response of double-opponent neurons reported in (Conway and Livingstone (2006), Fig. 15) and shown in Fig. 32

117 $$\begin{aligned}&h_\mathrm{double-opponent}(x_1, x_2, t;\; s, \tau )\nonumber \\&\quad = \pm (\partial _{x_1x_1} + \partial _{x_2x_2}) \, g(x_1, x_2;\; s) \nonumber \\&\qquad \times \partial _{{t'}^n} \, h(t;\; \tau ) \left( \begin{array}{c@{\quad }c@{\quad }c} \tfrac{1}{2} &{} - \tfrac{1}{2} &{} 0 \\ \tfrac{1}{2} &{} \tfrac{1}{2} &{} -1 \\ \end{array} \right) \end{aligned}$$

corresponding to an extension of (110) from purely spatio-chromatic image data to spatio-chrom-temporal image data. In the receptive fields measured by cell recordings, the rotational symmetry over the spatial domain is, however, not as fully developed for the spatio-chrom-temporal receptive fields as for the purely intensity-based spatial receptive fields.

### Motion selectivity

Concerning motion selectivity, DeAngelis et al. (1995), DeAngelis and Anzai (2004) report that most cortical neurons are quite *sensitive to stimulus velocity* and the speed tuning is more narrow than for LGN cells. Simple cells with inseparable receptive fields have directional preference while cells with space–time separable receptive fields do not. Moreover, the preferred direction of motion corresponds to the orientation of the filter in space–time.

This structure is nicely compatible with velocity adaptation, as described in Sects. 5.1 and 5.2. Within the above-mentioned terminology,

- *space–time separable* receptive fields correspond to spatio-temporal scale-space kernels without velocity adaptation, whereas
- *inseparable* receptive fields correspond to kernels that are explicitly adapted to nonzero velocities.

The directional preference of the cells in the spatial domain can, in turn, be controlled by the covariance matrix of the affine Gaussian scale-space concept as outlined in Sect. 3.2. We obtain receptive fields without directional preference in the spatial domain if we set the covariance matrix $\varSigma = s I$ proportional to the unit matrix, and space–time separable receptive fields if we in addition choose the velocity adaptation vector $v$ equal to zero. Assuming that the influence of $\varSigma$ and $v$ can be neglected (e.g., by setting $\varSigma$ proportional to the unit matrix and $v$ to zero), the filter shape will then be determined solely by the spatial scale $s$ and the temporal scale $\lambda$. Conversely, we can construct inseparable kernels with strong directional preference by appropriate combinations of the covariance matrix $\varSigma$ and the velocity adaptation vector $v$.

The above-mentioned fact that a majority of the cells are inseparable in space–time is indeed nicely compatible with a description in terms of a *multi-parameter scale space* as outlined in Sect. 2.1.3. If the vision system is to give a reasonable coverage of a set of filter parameters $\varSigma$ and $v$, then the set of filters corresponding to space–time separable receptive fields (corresponding to the filter parameters $v = 0$) will be much smaller than the set of filters allowing for nonzero values of the mixed parameters $\varSigma$ and $v$ over space and time.

### Complex cells

Besides the above-mentioned linear receptive fields, there is a large number of early *nonlinear* receptive fields that do not obey the superposition principle and whose response properties are rather insensitive to the phase of the visual stimuli. The response profile of such a cell in the spatial domain is typically of the form illustrated in Fig. 21c. Such cells for which the response properties are independent of the polarity of the stimuli are referred to as *complex cells* (Hubel and Wiesel 1959, 1962).

**Fig. 33:**
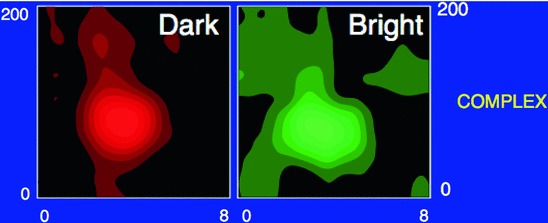
Response profile of a *complex cell* in the joint space–time domain as reported by DeAngelis et al. (1995), DeAngelis and Anzai (2004). Within the framework of the spatio-temporal scale-space framework presented in this paper, such a response property can be obtained by a quasi-quadrature combination of first- and second-order receptive fields; see Fig. 34 (*horizontal dimension*: space $x$, *vertical dimension*: time $t$)

In their study of spatio-temporal receptive field properties, DeAngelis et al. (1995), DeAngelis and Anzai (2004) also report a large number of complex cells with nonlinear response profiles in the joint space–time domain; see Fig. 33 for an example. Within the framework of the presented spatio-temporal scale-space concept, it is interesting to note that nonlinear receptive fields with qualitatively similar properties can be constructed by squaring first- and second-order derivative responses and summing up these components (Koenderink and Doorn 1990). Provided that the filters are appropriately normalized, we can then construct a *quasi-quadrature* measure over a one-dimensional either spatial or temporal domain as (Lindeberg 1997)

118 $$\begin{aligned} \mathcal{Q} L = L_{\xi }^2 + C \, L_{\xi \xi }^2 = s L_x^2 + C \, s^2 L_{xx}^2 \end{aligned}$$

where $\partial _{\xi } = \sqrt{s} \, \partial _x$ denotes *scale-normalized derivatives* with respect to scale-normalized coordinates $\xi = x/\sqrt{s}$ (Lindeberg 1998b) and where the constant $C$ can be determined either to minimize the amount of ripples in the operator response ($C = 2/3 \approx 0.667$) or from scale selection properties ($C = e/4 \approx 0.670$). Within this model, the first- and second-order Gaussian derivative approximations constitute an *approximation of a Hilbert pair* within the Gaussian derivative framework.

To extend this notion to a 1+1D space–time with receptive fields based on the Gaussian spatio-temporal scale-space concept, let us introduce normalized derivatives over scale-normalized time $\lambda = t /\sqrt{\tau }$ according to $\partial _{\lambda }\! =\! \sqrt{\tau } \, \partial _t$ or more generally $\partial _{\lambda }\! =\! \tau ^{\gamma /2} \, \partial _t$. Let us then define the following spatio-temporal generalizations of the quasi-quadrature measure

119 $$\begin{aligned}&\mathcal{Q}_1 L \!=\! L_{\xi }^2 \!+\! L_{\lambda }^2 \!+\! C \, (L_{\xi \xi }^2 \!+\! 2 L_{\xi \lambda }^2 \!+\! L_{\lambda \lambda }^2) \nonumber \\&\qquad \;\;\!=\! s L_{x}^2 \!+\! \tau L_{t}^2 \!+\! C \, (s^2 L_{xx}^2 \!+\! 2 s \tau L_{xt}^2 \!+\! \tau ^2 L_{tt}^2)\end{aligned}$$

120 $$\begin{aligned}&(\mathcal{Q}_2 L)^2 = (L_{\xi }^2 + C \, L_{\xi \xi }^2) (L_{\lambda }^2 + C \, L_{\lambda \lambda }^2) \nonumber \\&\qquad \qquad \; = (s L_{x}^2 + C \, s^2 L_{xx}^2) (\tau L_{t}^2 + C \, \tau ^2 L_{tt}^2)\end{aligned}$$

121 $$\begin{aligned}&\mathcal{Q}_3 L = L_{\xi \lambda }^2 \!+\! C \, L_{\xi \xi \lambda }^2 \!+\! C \, L_{\xi \lambda \lambda }^2 \!+\! C^2 \, L_{\xi \xi \lambda \lambda }^2 \nonumber \\&\qquad \quad \!=\! s \tau L_{xt}^2 \!+\! C \, s^2 \tau L_{xxt}^2 \!+\! C \, s \tau ^2 L_{xtt}^2 \!+\! C^2 \, s^2 \tau ^2 L_{xxtt}^2.\nonumber \\ \end{aligned}$$

For the time-causal scale-space, corresponding scale-normalized operators can be expressed as

122 $$\begin{aligned}&\mathcal{Q}_1 L = L_{\xi }^2 + L_{\lambda '}^2 + C \, (L_{\xi \xi }^2 + 2 L_{\xi \lambda '}^2 + L_{\lambda '\lambda '}^2) \nonumber \\&\qquad \quad = s L_{x}^2 + \tau L_{t'}^2 + C \, (s^2 L_{xx}^2 + 2 s \tau L_{xt'}^2 + \tau ^2 L_{t't'}^2)\end{aligned}$$

123 $$\begin{aligned}&(\mathcal{Q}_2 L)^2 = (L_{\xi }^2 + C \, L_{\xi \xi }^2) (L_{\lambda '}^2 + C \, L_{\lambda '\lambda '}^2) \nonumber \\&\qquad \qquad \,\, = (s L_{x}^2 + C \, s^2 L_{xx}^2) (\tau L_{t'}^2 + C \, \tau ^2 L_{t't'}^2)\end{aligned}$$

124 $$\begin{aligned}&\mathcal{Q}_3 L = L_{\xi \lambda '}^2 \!+\! C \, L_{\xi \xi \lambda '}^2 \!+\! C \, L_{\xi \lambda '\lambda '}^2 \!+\! C^2 \, L_{\xi \xi \lambda '\lambda '}^2 \nonumber \\&\qquad \;\; = s \tau L_{xt'}^2 \!+\! C \, s^2 \tau L_{xxt'}^2 \!+\! C \, s \tau ^2 L_{xt't'}^2 \!+\! C^2 \, s^2 \tau ^2 L_{xxt't'}^2\nonumber \\ \end{aligned}$$

where the temporal derivatives $\partial _{t'}$ with respect to self-similarly transformed time are related to derivatives with respect to regular time according to $\partial _{t'} \sim t^{\kappa } \partial _t$ and the exponent $\kappa$ should be in the interval $[0, 1]$ (Lindeberg 2011, Sect. 5.2).

**Fig. 34:**
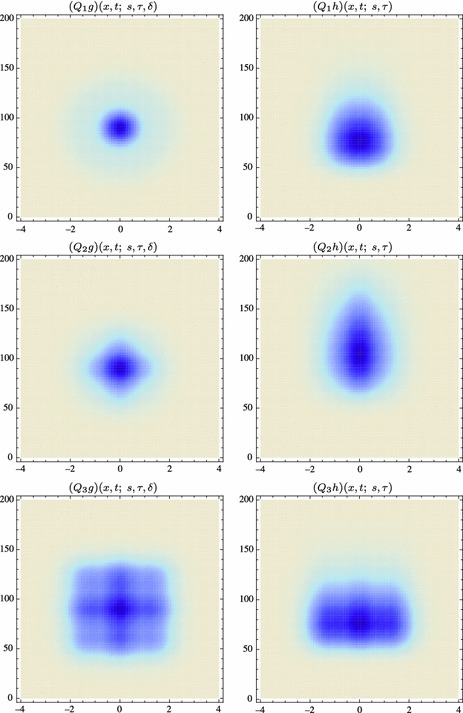
Idealized models of complex cells illustrated in terms of the response of different spatio-temporal quasi-quadrature measures to a delta function. (*left*) Computed for a spatio-temporal Gaussian $g(x, t;\; s, \tau , \delta )$ according to (*top*) $\mathcal{Q}_1 \, g = s \, g_{x}^2 + \tau g_{t}^2 + C \, (s^2 g_{xx}^2 + 2 s \tau g_{xt}^2 + \tau ^2 g_{tt}^2)$ (*middle*) $\mathcal{Q}_2^2 \, g = (s \, g_{x}^2 + s^2 g_{xx}^2) (\tau g_{t}^2 + C \, \tau ^2 g_{tt}^2)$ (*bottom*) $\mathcal{Q}_3 \, g = (s \tau g_{xt}^2 + C \, s^2 \tau g_{xxt}^2 + C \, s \tau ^2 \tau g_{xtt}^2 + C^2 \, s^2 \tau ^2 g_{xxtt}^2)$ with $s = 1.2, \tau = 25^2, \delta = 90, C = e/4$. (*right*) Computed for the time-causal kernel $h(x, t;\; s, \tau )$ according to (*top*) $\mathcal{Q}_1 \, h = s \, h_{x}^2 + \tau h_{t'}^2 + C \, (s^2 h_{xx}^2 + 2 s \tau h_{xt'}^2 + \tau ^2 h_{t't'}^2)$ (middle) $\mathcal{Q}_2^2 \, h = (s h_{x}^2 + s^2 h_{xx}^2) (\tau h_{t'}^2 + C \, \tau ^2 h_{t't'}^2)$ (*bottom*) $\mathcal{Q}_3 \, h = (s \tau h_{xt'}^2 + C \, s^2 \tau h_{xxt'}^2 + C \, s \tau 2 h_{xt't'}^2 + C^2 \, s^2 \tau ^2 h_{xxt't'}^2)$ with $s = 1.2, \tau = 25^2, \delta = 90, C = e/4$ (*horizontal dimension*: space $x$, *vertical dimension*: time $t$)

Figure 34 shows the result of computing the response of these quasi-quadrature measures to a delta function over a 1+1D space–time (without additional integration smoothing). Note that this type of computational structure is nicely compatible with results by Valois et al. (2000), who show that first- and second-order receptive fields typically occur in pairs that can be modelled as approximate Hilbert pairs. This model can therefore be interpreted as a Gaussian derivative-based analogue of the energy model for complex cells proposed by (Adelson and Bergen 1985; Heeger 1992).

As a complement to the above *pointwise* computation quasi-quadrature entities, we can apply a second-stage smoothing step

125 $$\begin{aligned}&(Q' L)(x, t;\; \varSigma _\mathrm{der}, \varSigma _\mathrm{int})\nonumber \\&\quad = \int \limits _{(u, v) \in {\mathbb R}^2 \times {\mathbb R}} (Q L)(x\!-\!u, t\!-\!v;\; \varSigma _\mathrm{der}) \, h(u, v;\; \varSigma _\mathrm{int}) \, \hbox {d}u \, \hbox {d}v\nonumber \\ \end{aligned}$$

with convolution kernel $h_\mathrm{int}(\cdot , \cdot ;\; \varSigma _\mathrm{int})$ over space or space–time with integration scale $\varSigma _\mathrm{int}$ and with $\varSigma _\mathrm{der}$ denoting the regular local scale parameter for computing derivatives. For the quasi-quadrature entities derived from the Gaussian spatio-temporal scale-space, we should of course choose a non-causal Gaussian spatio-temporal kernel, whereas we should for the corresponding entities derived from the time-causal spatio-temporal scale-space choose a time-causal spatio-temporal kernel for the second-stage integration smoothing. Computationally, such a second-stage smoothing step can be performed with similar diffusion mechanisms as used for performing the first stage of spatial and/or temporal scale-space smoothing. With such an additional post-smoothing stage, the response properties of these quasi-quadrature cells will be rather insensitive to the phase of the visual input and do in this respect agree with the approximate phase invariance of complex cells noted by Hubel and Wiesel (1959, 1962).

In a detailed study of the response properties of complex cells, Touryan et al. (2002) observed an additive interaction between the eigenvectors of a quadratic nonlinear model supporting the energy model (Adelson and Bergen 1985; Heeger 1992). In a more recent study, Rust et al. (2005) found that complex cell responses are better described by more linear filters than the one or two used in previous models. The above-mentioned quasi-quadrature models are in qualitative agreement with such computational structures. Specifically, the second-stage smoothing (125) of the pointwise quasi-quadrature measure is in good agreement with the model of complex cell responses in (Rust et al. (2005), Fig. 8, page 953) based on weighted averaging of a set of quadrature pairs.

Cell recordings have indicated that receptive fields may also be affected from stimuli outside the support region of the classical receptive field (Cavanaugh et al. 2001a, b) and that non-optimal stimuli, e.g., of different orientations than the tuning of the cell, may lead to a suppressive influence on the response properties of complex cells (Ringach et al. 2002; Rust et al. 2005; Felsen et al. 2005). Such suppressive influence can be obtained by (i) complementing the quasi-quadrature model with *divisive normalization* ( Heeger 1992; Schwartz and Simoncelli 2001) with respect to an ensemble of different nonlinear feature detectors $\mathcal{Q}_i L$ with their respective weights $w_i$ according to

126 $$\begin{aligned} r = \frac{\mathcal{Q} L}{\sum _i w_i \, \mathcal{Q}_i L + c^2}. \end{aligned}$$

With the quasi-quadrature entities $\mathcal{Q}_i L$ defined from spatio-temporal receptive fields with directional tuning in the spatial domain given by a spatial covariance matrix $\varSigma _i$, an image velocity $v_i$ and a temporal scale $\tau _i$

127 $$\begin{aligned} (\mathcal{Q}_i L)(x, t) = (\mathcal{Q}_i L)(x, t;\; \varSigma _i, v_i, \tau _i) \end{aligned}$$

an *ensemble* of such nonlinear receptive fields would then correspond to a *population coding* over different spatial orientations, motion directions, and temporal scales.

If we assume that the feature detector $F_0$ at the center $x_0$ of the receptive field is tuned to a special orientation $\theta _0$ as determined by a covariance matrix $\varSigma _0$ in space, to an image velocity $v_0$ in space–time, and to a temporal scale $\tau _0$, then the stimulation of another feature detector $F_i$ at a neighboring spatial position $x_i$ tuned to an orientation $\theta _i$ as determined by a covariance matrix $\varSigma _i$ in space, image velocity $v_i$, and temporal scale $\tau _i$ may suppress the output of $F_0$ depending on the relationships between $\varSigma _i$, $\varSigma _0$, $v_i$, $v_0$, $\tau _i$, and $\tau _0$. An interesting question concerns whether the weights $w_i$ can be determined from these entities based on geometric relationships

128 $$\begin{aligned} w_i = F(x_i, x_0, \varSigma _i, \varSigma _0, v_i, v_0, \tau _i, \tau _0) \end{aligned}$$

or whether some other nonlinear model would be preferable. To investigate this issue, more experimental data would be needed.

Suppressive influence can also be obtained by allowing for (ii) *nonlinear feedback* that alters the conductivities in the diffusion equation (112) alternatively the corresponding spatio-temporal extension based on local image measurements or by considering (iii) recurrent feedback from higher levels that influence the gain control of the feature detectors. With these extensions, the resulting model corresponds to an integration of a hierarchical and recurrent models as advocated by Martinez and Alonso (2003).

In contrast to the previous treatment of linear receptive field models, which were determined by necessity from first principles, it should be emphasized that the structure of the quasi-quadrature model is not at all determined by necessity. Instead, it is presented as one possible nonlinear extension that reproduces some of the qualitative properties of complex cells.

## Foveated vision

**Fig. 35:**
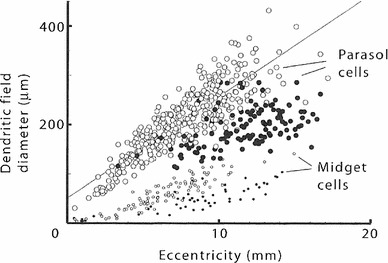
Results of measurements of the receptive field size as a function of eccentricity for ganglion cells in the retina from Martin and Grünert (2004) based on the results by Watanabe and Rodieck (1989). The parasol cells project to the magnocellular pathway (corresponding to motion perception), whereas the midget cells project to the parvocellular pathway (corresponding to shape perception)

Concerning the assumption of translational invariance over the spatial domain, it is well known that the retina of humans and other higher mammals is not translationally invariant. Instead, finer scale receptive fields are concentrated toward a *fovea* in such a way that the spatial extent of the receptive fields *increases essentially linearly with eccentricity* (Koenderink and Doorn 1978) (see Fig. 35).

**Fig. 36:**
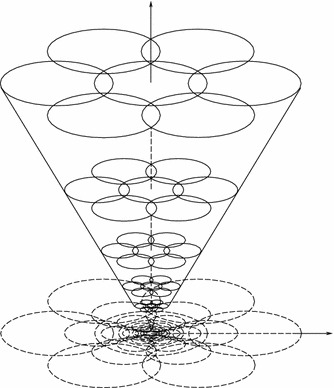
Foveal scale-space model as obtained from the complementary assumptions of (i) a *finite processing capacity* that is to be uniformly distributed over scales and (ii) a *preferred image point* whose location can be shifted by a focus-of-attention mechanism to simulate full translational invariance

There are close similarities between such a behavior and the distribution of receptive fields that is obtained if we assume that the visual system has a *limited processing capacity* that is to be distributed over receptive fields at different scales. If we assume that the idealized vision system has a *focus-of-attention* mechanism that allows it to simulate translation invariance by changing the viewing direction, then based on the argument of scale invariance, it is natural to distribute the limited processing capacity in such a way that a *similar amount of processing capacity* is available *for all scales* within some scale range $[s_{\min }, s_{\max }]$. In other words, the vision system should have the same number of receptive fields at all scales within some finite scale range (see Fig. 36).

Given these assumption, it follows that *the minimum receptive field size will increase linearly with the distance from the fovea*, a distribution that is compatible with neurophysiological and psychophysical findings (Lindeberg and Florack 1992). Given such a spatially varying resolution limit, internal representations at coarser scales can then be constructed from these image measurements based on the semigroup property or the diffusion equation. Specifically, with a *log-polar retinotopic mapping*, the diffusion equation that governs the evolution properties over scale can equivalently be expressed on a log-polar domain (Lindeberg and Florack 1994). In all other respects, the receptive field profiles will be similar as for a translationally invariant spatial domain.

This foveal scale-space model has been used for computing scale-invariant image descriptors for object recognition by Kokkinos and Yuille (2008). A closely related model for foveal vision in terms of an inverted pyramid has been proposed by Crowley and his co-workers (1994) with close relations to the spotlight model for visual attention by Tsotsos (1995).

A notable property of the receptive field measurements taken in the retina as shown in Fig. 35 is that the receptive field sizes are clustered along linear functions, whereas the foveal scale-space model in Fig. 36 is based on the assumptions that all receptive field sizes above a linearly increasing minimum receptive field size should be present. Given the semigroup property (8), it follows, however, that receptive fields at scales coarser than those displayed in Fig. 35 can be constructed by combining receptive fields at finer scales. The distribution in Fig. 35 would therefore correspond to a sampling of the *outer layer* of the inverted cone of receptive field sizes in the foveal scale-space model shown in Fig. 36. Receptive fields in the interior of this cone can therefore be constructed from linear combinations of receptive field responses in the outer layer.

An interesting question concerns whether the existence of coarser-scale receptive fields corresponding to the interior of this cone could be established by cell recording of linear receptive fields in the LGN or in V1. An alternative possibility could be to investigate whether receptive fields corresponding to the outer layer of this cone could be directly combined into nonlinear receptive fields corresponding to the interior of this cone, without representing the intermediate linear receptive fields explicitly in terms of simple cells. Such investigations could then answer whether and how shift invariance is explicitly represented at the earliest levels of linear receptive fields or at higher nonlinear levels in the visual hierarchy.

## Extensions

With regard to camera geometry, we have throughout based the analysis on a planar perspective projection model with a flat image plane. This choice has been made to simplify the mathematical treatment, since the translational group properties and the diffusion equations are much easier to express for a flat image geometry. To model biological vision more accurately, it would, however, be more appropriate to express a corresponding model based on a *spherical camera geometry* with a spherical image surface, which will lead to a scale-space concept based on diffusion equations on a sphere. Such a model would also have attractive theoretical properties in the sense that geometric distortions toward the periphery, such as vignetting, will disappear, and certain properties of global motion fields will become simpler. From such a background, the present model can be regarded as a *local linearization* applied in the tangent plane of the spherical camera model at the center of the visual sensor.

With regard to the logarithmic transformation of the intensity domain, it is also worth emphasizing that if we have an initial visual sensor that compresses the brightness range according to a *self-similar intensity transformation* $I' = I^{\gamma }$ with $\gamma < 1$, then the result of applying a logarithmic transformation to this output

129 $$\begin{aligned} f(x) = \log I^{\gamma }(x) = \gamma \, \log I(x) \end{aligned}$$

will be of a similar form as of applying a corresponding transformation to the original data, with the only difference that the range of variations for the corresponding receptive fields will be compressed by a uniform factor $\gamma < 1$ (gamma compression). In this respect, the presented model might find interesting applications when constructing computational models of human vision for evaluating the perceptual quality of image displays.

## Relations to previous work

### Biological vision

The notion of receptive field was originally defined by Sherrington (1906) to describe the somatosensory area of a body surface where a stimulus could cause a reflex. Hartline (1938) extended this notion to light stimuli and defined a *visual receptive field* as the area of the retina that must receive illumination in order to cause a discharge in a particular ganglion cell or nerve fiber. Kuffler (1953) studied the substructure of retinal receptive fields and found that they are concentric with specific “on” or “off” zones. He also coined the term “on–off” receptive fields. The Nobel laurates Hubel and Wiesel (1959, 1962, 2005) investigated and characterized the response properties of cells in the primary visual cortex (V1), discovered their orientation tuning, and proposed a taxonomy in terms of simple or complex cells based on how the cells respond to the polarity of visual stimuli. In the first wave of studies, specific stimuli such as points, bars, or sine wave gratings were used as stimuli for probing the visual cells.

Later, a new methodology for receptive field mappings was developed based on white noise stimuli, which allow for a complete characterization of the response properties of visual neurons if they can be assumed to be linear. Based on this technique, DeAngelis et al. (1995) were able to derive more detailed maps of receptive fields, including their response properties in the *joint* space–time domain; see DeAngelis and Anzai (2004) for a comprehensive overview of these developments. Conway and Livingstone (2006) performed a corresponding investigation of spatio-chromatic and spatio-chrom-temporal response properties of receptive fields in the macaque monkey. Ringach et al. (2002) showed how receptive field profiles of neurons can be derived using natural image sequences as stimuli. Felsen et al. (2005) have presented comparisons between response properties of neurons to natural image features versus noise stimuli and found that in the responses of complex cells, but not of simple cells, the sensitivity is markedly higher for natural image data than for random stimuli.

Adelson and Bergen (1985) developed a spatio-temporal energy model for motion perception based on oriented filters in the space–time domain. The quasi-quadrature approach in (118) and (119) in combination with a multi-parameter scale space can be seen as an analogue and extension of such a representation within the Gaussian derivative framework. More recently, Young et al. (2001) showed how spatio-temporal receptive fields can be modelled by Gaussian derivatives over a spatio-temporal domain, corresponding to the Gaussian spatio-temporal concept described here, although with a different type of parameterization.

The scale-space models described in this article and our earlier work (Lindeberg 1997, 2001, 2011) *unify* these treatments into a joint framework and do also comprise new extensions in the following ways: (i) a *new continuous time-causal scale-space model* that respects forbidden access to future information, (ii) a *time recursive* update mechanism based on a limited temporal buffer, (iii) a *better parameterization* of the spatio-temporal filters with respect to image velocities and image deformations, and (iv) *necessity results* showing how these scale-space models can be uniquely determined from a small set of structural assumptions regarding an idealized vision system.

It should be emphasized, however, that the theoretical necessity results presented in this paper concern *linear* receptive fields. Characterizing nonlinear receptive fields is a much more complex issue, see Ringach (2004) for an overview of different approaches for mapping receptive fields. Nonlinear gain control mechanisms in the retina have been modelled and related to biological cell recordings by Schwartz et al. (2002). Nonlinear receptive fields in V1 have been investigated and modelled in more detail by Mechler and Ringach (2002), Touryan et al. (2002), Priebe et al. (2004), and Rust et al. (2005). During recent years, there has been some questioning of whether the taxonomy by Hubel and Wiesel into simple and complex cells corresponds to distinct classes or whether V1 cells have response properties along a continuum (Mechler and Ringach 2002). Bardy et al. (2006) have shown that the response properties of some classes of complex cells can be converted to putative simple cells depending on influences originating from the classical receptive field. The experimental results can, however, be strongly dependent on the experimental conditions (Kagan et al. 2002; Mata and Ringach 2005; Chen et al. 2002) and bimodal distributions have been found by Kagan et al. (2002), Ibbitson et al. (2005), and Chen et al. (2002). Moreover, Martinez and Alonso (2003) argue that a large body of neurophysiological evidence indicates that simple cells are a separate population from the total of cortical cells in cat visual cortex. In relation to the classification of complex cells, Kagan et al. (2002) have suggested that distinctions in the classification of complex cells should be made on whether the cells are dominated by magnocellular or parvocellular input. Martinez and Alonso (2003) have suggested that complex cells should be divided into first-order complex cells that receive direct input from the LGN and second-order complex cells that receive input from simple cells. More recently, Williams and Shapley (2007) have found spatial phase-sensitive detectors in V1 that respond to contrast boundaries of one sign but not the opposite. Our knowledge about nonlinear cells in area V1 is therefore far from complete (Olshausen and Field 2004; Carandini et al. 2005).

The notion of a *logarithmic brightness scale* goes back to the Greek astronomer Hipparchus, who constructed a subjective scale for the brightness of stars in six steps labelled “1 ...6,” where the brightest stars were said to be of the first magnitude ($m = 1$) while the faintest stars near the limits of human perception were of the sixth magnitude. Later, when quantitative physical measurements were made possible of the intensities of different stars, it was noted that Hipparchus subjective scale did indeed correspond to a logarithmic scale. In astronomy today, the *apparent brightness* of stars is still measured on a logarithmic scale, although extended over a much wider span of intensity values. A logarithmic transformation of image intensities is also used in the retinex theory (Land 1974, 1986).

In psychophysics, the *Weber-Fechner law* attempts to describe the relationship between the physical magnitude and the perceived intensity of stimuli. This law states that the ratio of an increment threshold $\Delta I$ for a just noticeable difference in relation to the background intensity $I$ is constant over large ranges of magnitude variations (Palmer 1999, pages 671–672)

130 $$\begin{aligned} \frac{\Delta I}{I} = k \end{aligned}$$

where the constant $k$ is referred to as the Weber ratio. The theoretical analysis of invariance properties of a logarithmic brightness scale under multiplicative transformations of the illumination field as well as multiplicative exposure control mechanisms is in excellent agreement with these psychophysical findings.

For a strictly positive entity $z$, there are also information theoretic arguments to regard $\log z$ as a default parameterization (Jaynes 1968). This property is essentially related to the fact that the ratio $dz/z$ then becomes a dimensionless integration measure. A general recommendation of care should, however, be taken when using such reasoning based on dimensionality arguments, since important phenomena could be missed, e.g., in the presence of hidden variables. The physical modelling of the effect on illumination variation on receptive field measurements in Sect. 2.3 provides a formal justification for using a logarithmic brightness scale in this context as well as an additional contribution of showing how the receptive field measurements can be related to inherent physical properties of object surfaces in the environment.

### Computer vision

In the area of computer vision, multi-scale representations were first constructed by repeated smoothing and subsampling, leading to the notion of *pyramids* (Burt 1981; Crowley 1981; Burt and Adelson 1983; Crowley and Stern 1984; Crowley and Parker 1984; Crowley and Sanderson 1987).

Concerning the development of *scale-space theory*, Witkin (1983) proposed to treat scale as a continuous parameter and noted that Gaussian convolution leads to a decreasing number of zero-crossings or local extrema for a one-dimensional signal. The first necessity results in the Western literature concerning the uniqueness of the Gaussian kernel for generating a linear scale-space representation were derived by Koenderink (1984) based on the assumption of *causality*, which means that iso-surfaces in scale space should point with their convex side toward coarser scales. Related uniqueness results were presented by Babaud et al. (1986) and by Yuille and Poggio (1986).

Lindeberg (1990) showed how a reformulation of Koenderink’s causality requirement in terms of *non-enhancement of local extrema* in combination with the requirement of a semigroup structure could be used for deriving a scale-space theory for discrete signals. Corresponding necessity results concerning scale-space representations of continuous image data based were then presented in Lindeberg (1996). A cascade property was also used in the construction of binomial pyramids by Crowley (1981), Crowley and Stern (1984).

Florack and Haar Romeny (1992) proposed to the use of *scale invariance* as a basic scale-space axiom and Pauwels et al. (1995) showed that in combination with a semigroup structure, there exists a more general one-parameter family of (weak) scale-space kernels that obey these axioms, including the Poisson scale space studied by Felsberg and Sommer (2004), Duits et al. (2004) have investigated the properties of these scale spaces in detail and showed that the so-called $\alpha$-scale spaces can be modelled by pseudo-partial differential equations. Except for the Gaussian scale space contained in this class, these *self-similar scale spaces* do, however, not obey non-enhancement of local extrema.

Closely related axiomatic derivations of image processing operators based on scale invariance have also been given in the earlier Japanese literature (Iijima 1962; Weickert et al. 1999). Koenderink and Doorn (1992) showed that Gaussian derivative operators are natural operators to derive from a scale-space representation, given the assumption of scale invariance.

The connections between the strong regularizing properties of Gaussian convolution with Schwartz distribution theory have been pointed out by Florack et al. (1992).

Generalizations of rotationally symmetric smoothing operations to the *affine Gaussian scale-space* concept were introduced in (Lindeberg 1994b) and applied in (Lindeberg and Gårding 1997) for the computation of affine invariant image descriptors. Specifically, a mechanism of *affine shape adaptation* was proposed for reaching affine covariant interest points in affine scale space, and it was shown that the computation of such affine-adapted image measurements improved the accuracy of later-stage processes in situations when there are significant perspective image deformations outside the similarity group. Baumberg (2000) and Schaffalitzky and Zisserman (2001) furthered this approach to wide baseline image matching. Mikolajczyk and Schmid (2004) proposed a more efficient algorithm and quantified its performance experimentally. Tuytelaars and Gool (2004) performed corresponding matching of widely separated views with application to object modelling. Related investigations of elongated directional filters over the spatial domain have been presented by Freeman and Adelson (1991); Simoncelli et al. (1992) and Perona (1992).

Scale-space representations of *color information* have been developed by Geusebroek et al. (2001) based on a Gaussian color model proposed by Koenderink, from which a set of differential color invariants were defined and by Hall et al. (2000) who computed first-order partial derivatives of color-opponent channels and demonstrated the applicability of such features for object recognition. Linde and Lindeberg (2004, 2012) extended this idea by showing that highly discriminative image descriptors for object recognition can be obtained from spatio-chromatic derivatives and differential invariants up to order two. More recently, Sande et al. (2010) have presented an evaluation of different color-based image descriptors for recognition.

Concerning *temporal scale spaces*, Koenderink (1988) proposed the first scale-space concept that respects temporal causality, based on a logarithmic transformation of the time axis with the present moment as the origin. Such temporal smoothing filters have been considered in follow-up works by Florack (1997) and ter Haar Romeny et al. (2001). These approaches, however, appear to require infinite memory of the past and have so far not been developed for computational applications.

To handle time causality in a manner more suitable for real-time implementation, Lindeberg and Fagerström (1996) expressed a strictly time-recursive space–time separable spatio-temporal scale-space model based on the cascades of temporal scale-space kernels in terms of either truncated exponential functions or first-order recursive filters, based on a characterization of one-dimensional scale-space filters that guarantee non-creation of local extrema with increasing scale (Lindeberg 1990). These scale spaces were also *time recursive* in the sense that no extensive memory of the past was needed. Instead, a compact temporal buffer allowed for efficient computation of the temporal smoothing operation and temporal derivatives directly from a set of internal representations at different temporal scales. A closely related time-recursive computation of temporal derivatives has been used by Fleet and Langley (1995).

Lindeberg (1997) proposed a non-separable *spatio-temporal scale-space* concept comprising the notion of *velocity-adapted derivatives* for a continuous model based on a Gaussian spatio-temporal scale-space and for a semi-discrete time-causal model; see also Lindeberg (2001) for a more detailed description of the corresponding spatio-temporal scale-space theory. Velocity adaptation was applied to optic flow estimation by Nagel and Gehrke (1998) and was shown to improve the accuracy in optic flow estimates in a similar manner as affine shape adaptation improves the accuracy of image descriptors under perspective image deformations outside the similarity group. A closely related approach for optic flow computation with corresponding deformation of the image filters was developed by Florack et al. (1998). An extension of non-separable spatio-temporal fields into time-causal velocity-adapted recursive filters was given in (Lindeberg 2002).

Laptev and Lindeberg (2004b) investigated the use of families of velocity-adapted filters for computing *Galilean invariant image descriptors*. Given an ensemble of spatio-temporal scale-space filters with different orientations in the space–time domain in a manner similar to Adelson and Bergen (1985), simultaneous adaptation to spatial scales, temporal scales, and image velocities was performed by a multi-parameter scale selection mechanism over these parameters. Specifically, it was shown that the use of velocity-adapted filters improved the separability between classes of spatio-temporal actions in situations when there are unknown relative motions between the objects and the observer. Generalizations of this approach to the context of Galilean invariant interest points were then presented in Lindeberg (2004) with an integrated Galilean invariant spatio-temporal recognition scheme in (Laptev et al. 2007).

Fagerström (2005) investigated self-similar temporal scale-space concepts derived from the assumptions of a semigroup structure combined with scale invariance, with an extension to the spatio-temporal domain in Fagerström (2007) that also comprises the notion of velocity-adapted filters. Lindeberg (2011) gives a unified treatment of the scale-space axiomatics of linear, affine, and spatio-temporal scale space for continuous images based on the assumption of non-enhancement of local extrema over spatial and spatio-temporal domains, including more explicit statements of the uniqueness results regarding the Gaussian spatio-temporal scale space earlier outlined in Lindeberg (2001) and the application of non-enhancement of local extrema to a continuous time-causal and time-recursive spatio-temporal scale space.

## Summary and conclusions

Neurophysiological recordings have shown that mammalian vision has developed receptive fields that are tuned to different sizes and orientations in the image domain as well as to different image velocities in space–time. A main message of this article has been to show that it is possible to derive such families of receptive field profiles *by necessity*, given a set of structural requirements on the first stages of visual processing as formalized into the notion of an *idealized vision system*. These structural requirements reflect *structural properties of the world* in terms of scale covariance, affine covariance, and Galilean covariance, which are natural to *adapt to* for a vision system that is to *interact with the surrounding world* in a successful manner. In a competition between different organisms, adaptation to these properties may constitute an *evolutionary advantage*.

The presented theoretical model provides a *normative theory* for deriving *functional models of linear receptive fields* based on Gaussian derivatives and closely related operators. In addition, a set of plausible mechanisms have been presented of how nonlinear receptive fields can be constructed from this theory, based on a generalized energy model. Specifically, the proposed theory can *explain* the different shapes of receptive field profiles that are found in biological vision from a requirement that the visual system should be able to compute covariant receptive field responses under the natural types of image transformations that occur in the environment, to enable the computation of invariant representations for perception at higher levels.

The proposed receptive field model has been related to Gabor functions, and we have presented several theoretical arguments for preferring a Gaussian derivative model or equivalently a formulation in terms of *diffusion equations*, with the shapes of the receptive fields parameterized by a spatial covariance matrix $\varSigma$, an image velocity $v$ and a temporal scale parameter $\tau$, where the spatial covariance matrix $\varSigma$ can also encompass the spatial scale parameter $s$ depending on the choice of parameterization.

In the most idealized version of the theory, one can see the covariance matrix $\varSigma$ in the diffusion equation and the image velocity $v$ as locally constant within the support region of each receptive field, corresponding to a pure feed-forward model. More generally, one can consider covariance matrices and image velocities that are locally adapted to the local image structures, leading to richer families of pseudo-linear or nonlinear scale spaces, corresponding to top-down or feedback mechanisms in biological vision.

When the image data undergo natural image transformations due to variations in viewing distance, viewing direction, relative motion between the object and the observer or illumination variations, we can linearize the possibly nonlinear image transformations locally by derivatives (Jacobians), from which transformation properties in terms of the filter parameters (scale parameters, covariance matrices, and image velocities) of the receptive fields can be derived, provided that the family of receptive fields is closed under the relevant group or subgroup of image transformations in the tangent space, leading to an algebra of transformation properties of receptive fields. In this article, we have presented a coherent and unified framework for handling such locally linearized image transformations in terms of local scaling transformations, local affine transformations, local Galilean transformations, and local multiplicative intensity transformations, such that the influence of these image transformations on the receptive field responses can be well understood. More generally, the formulation of image primitives in terms of receptive field responses that are expressed in terms of scale-space derivatives makes it possible to use tools from differential geometry for deriving relationships between image features and physical properties of objects or events in the environment, thus allowing for computationally operational and theoretically well-founded modelling of possibilities or constraints for visual perception.

We have also related the proposed approach to approaches for learning receptive field profiles from *natural image statistics* and argued that the presented model in such a context provides a normative theory for the solutions that an idealized learning system may reach if exposed to a sufficient large and representative set of natural image data. The presented theory can therefore be used for explaining why such learning approaches lead to qualitatively similar types of receptive fields.

Several of the theoretically derived receptive field profiles presented in this article have been successfully used in a large number of computer vision applications regarding feature detection, feature classification, stereo matching, motion estimation, shape analysis, and image-based recognition. Hence, these receptive field profiles can generally serve as a basis for expressing a *large number of visual operations* and have empirically been shown to lead to robust algorithms. In this respect, a vision system based on these receptive field families allows for *sharing* of early visual modules between different higher level vision functionalities, which for a biological vision system can be motivated by efficiency of resource utilization.

The linear receptive fields obtained from this theory have been compared to receptive fields found by cell recordings in the LGN and simple cells in V1.

The proposed nonlinear quasi-quadrature model has also been related to qualitatively similar properties observed for complex cells in V1.

A striking conclusion from the comparisons in Sect. 6 is that the receptive field profiles derived by the *axiomatic theory* in Sects. 3–5 are in *very good qualitative agreement* with receptive field profiles recorded in *biological vision*. Thus, we have a very good match between consequences of the theory and experimental data.

Furthermore, this indicates that the earliest receptive fields in higher mammal vision have reached a state that is very close to *ideal* in view of the stated structural requirements or symmetry properties. In this sense, biological vision can be seen as having adapted very well to the transformation properties of the surrounding world and the transformations that occur when a three-dimensional world is projected onto a two-dimensional image domain.

### Applications to biological vision

The presented theory provides a *theoretically well-founded computational model* for early receptive fields. We propose that this theory could be used as a powerful and general tool for modelling biological vision, at least in the following ways:

- The Gaussian and the time-causal receptive field families with their spatial and spatio-temporal derivative operators applied to luminance and color-opponent channels can be used for generating wider and more general families of receptive field profiles beyond those explicitly shown in the figures in this article. The idealized model for simple cells (116) comprises receptive fields of different orders of spatial and temporal differentiations, where a subset of combinations of spatial and spatio-temporal derivative operators has been demonstrated to lead to receptive field profiles in good qualitative agreement with receptive field profiles measured by cell recordings in biological vision. An interesting question concerns whether the existence of linear receptive fields corresponding to other combinations of spatial and spatio-temporal derivatives can be demonstrated, in particular when the receptive fields are measured as functions over two spatial dimensions and one temporal dimension and concerning the existence of receptive fields corresponding to higher orders of derivatives. Concerning spatio-chromatic and spatio-chrom-temporal receptive fields, the models for double-opponent receptive fields (110) and (117) are both based on rotationally symmetric Laplacians of Gaussians (alternatively differences of Gaussians) concerning the spatial dependencies. Another interesting question concerns whether biological vision implements non-symmetric spatio-chromatic receptive fields corresponding to, e.g., directional or partial derivatives of color-opponent channels as shown in Fig. 9, and whether or whether not tighter couplings could be established between the chromatic and temporal dimensions. Answering these questions would provide cues to what types of image structure the visual system explicitly responds to and therefore possibilities as well as limitations for perception. Hence, this theory may be used for generating *predictions* about new hitherto unnoticed or unreported receptive fields and for explaining their properties in terms of differential geometric measurements. This theory can also be used for raising questions about which animals have early receptive fields with properties compatible with general purpose visual operations according to the notion of an idealized visual front end.
- Concerning orientation maps and population coding over image orientations and image velocities, the notion of multi-parameter receptive field families over different spatial covariance matrices $\varSigma$, image velocities $v$, and temporal scales $\tau$ raises questions of how the receptive fields in V1 are distributed over different orientations and directional tunings. Since receptive fields have been found with different degrees of spatial eccentricities, corresponding to different scale parameters in different directions, this raises questions of whether the distribution over different degrees of spatial elongation is such that it could be explained by a geometric model over spatial covariance matrices $\varSigma _i$ corresponding to structural properties of the environment. More generally and as we have previously discussed in Sect. 6.6, given that we have a population of nonlinear receptive fields that are tuned to different spatial orientations and motion directions that respond according to an energy model, an interesting question concerns how to combine the responses of a set of such nonlinear receptive fields that respond at different spatial locations and tuned to different orientations and motion directions. Could a sufficient amount of cell recordings be gathered to answer the question of how this information should be combined from a population of such nonlinear detectors, e.g., for setting the relative weights for divisive normalization or by changing the conductivities in the diffusion equations that determine the properties of the underlying receptive fields. In connection with the foveal scale-space model in Sect. 7 and the dominance of receptive fields with a linearly increasing receptive field size as function of eccentricity found by cell recordings of retinal ganglion cells, it would also as discussed in at the end of Sect. 7 be interesting to know whether and where the existence of coarser-scale receptive fields corresponding to the interior of the inverted cone in Fig. 36 could be established. In these and other ways, the presented mathematical framework for receptive fields could be used for expressing and raising questions about computational mechanisms.
- The theoretical covariance properties of the associated scale-space concepts allow for *explicit handling of invariance properties* with respect to scale variations, image deformations, and relative motions. In computational models, such as neural networks, explicit incorporation of such transformation properties may be used for *bypassing* the need for an explicit *training stage* to learn corresponding invariance properties. From a biological standpoint, it appears natural that biological organisms should develop the possibility of having these transformations hard-wired or soft-wired (the latter notion meaning that a set of initial connections being trimmed after birth), since these transformations are universal. In terms of receptive fields, these transformations will then correspond to certain parameter ranges of the scale parameters, determined by the statistics of natural images. This theory may therefore be more generally used for reducing or bypassing the need for explicit learning the spatial, spatio-chromatic, and spatio-temporal response properties of early receptive fields in computational models of visual perception. In this respect, the presented theory could allow for *lower needs for training data* and a lower amount of *computational resources* in the training stage of computational vision models, by faster formation of receptive fields given a hard-wired or soft-wired architecture. The theory may also imply higher *robustness* of early receptive fields in computational models and require *less variability* in the training data.
- With regard to a possible biological implementation of this theory, the evolution properties of the presented scale-space models are governed by *diffusion equations*, which can be implemented by *operations over neighborhoods*. Hence, the computations can naturally be implemented in terms of *connections between different cells*. Diffusion equations are also used in mean field theory for approximating the computations that are performed by populations of neurons (Omurtag et al. 2000; Mattia and Guidice 2002; Faugeras et al. 2009). The generalized semigroup property (8) with the corresponding cascade property (9) possibly expressed for a multi-parameter scale space and the diffusion equations in terms of infinitesimal generators (13) and (14) describe how receptive fields corresponding to different possibly multi-dimensional scale parameters can be *related* and hence how receptive fields at coarser scales can be computed from receptive fields at finer scales. In a neural network implementation, these relations can hence be used for *setting the weights* for communications between different cells. This theory also provides a framework for modelling and explaining the temporal dynamics of neural computations between cells at different levels of processing. In this respect, the theory naturally leads to a *hierarchical architecture* with explicit expressions for how receptive fields in the fovea can constitute the basis for receptive fields in the LGN and these in turn can be used for defining receptive fields in V1 and later stages in the visual cortex.

It should be emphasized, however, that this model has not been primarily constructed to accurately reproduce experimental findings regarding biological vision. Instead, the focus has been on formulating an *idealized theoretical model* for the types of computations that are natural to perform at the *earliest stages of visual processing* given theoretical properties of the structure of the surrounding world, which are then expressed as fundamental assumptions about the functionality of the vision system. If the model should be regarded as *biomimetic*, that would then be in a weaker sense of performing similar types of functions.

In this way, specific properties of specific organisms are suppressed (and not considered here because of reasons of scope). The approach is therefore more related to approaches in *theoretical physics*, where symmetry properties of the world are used as fundamentals in the formulation of physical theories. In the area of scale-space theory, these structural assumptions are referred as *scale-space axioms*.

## Footnotes

1 Concerning notation, we will throughout use a notation similar to physics or mathematics, with scalars and vectors represented by lower case letters, $a \in {\mathbb R}$ and $x \in {\mathbb R}^2$, (without explicit notational overhead for vectors) and matrices represented by upper case letters, $A$ or $\varSigma$. Operators that act on functions will be represented by calligraphic symbols, $\mathcal{T}$ and $\mathcal{A}$, and we use either lower case or upper case letters for functions, $f$ and $L$. The overall convention is that the meaning of a symbol is defined the first time it is used.

2 In Eq. (1), the symbol “$\cdot$” at the position of the first argument of $L$ is a place holder to emphasize that in this relation, $L$ is regarded as a function and not evaluated with respect to its first argument $x$. The following semi-colon emphasizes the different natures of the image coordinates $x$ and the filter parameters $s$.

3 More precisely, we will assume that linearity should hold for some transformation $f = z(I)$ of the original luminosity values $I$ in units of local energy measurements. In Sect. 2.3 it will be shown that a logarithmic intensity mapping $f \sim \log I$ is particularly attractive in this respect by allowing for invariance of receptive field responses under local multiplicative intensity transformations.

4 For us humans and other higher mammals, the retina is obviously not translationally invariant. Instead, finer scale receptive fields are concentrated to the fovea in such a way that the minimum receptive field size increases essentially linearly with eccentricity (see Sect. 7). With respect to such a sensor space, the assumption about translational invariance should be taken as an idealized model for the region in space where there are receptive fields above a certain size.

5 The symbol “$\cdot$” used as placeholder for the first argument of $T$ and the argument of $f$ in Eq. (4) indicate that the convolution operation “$*$” is performed over the corresponding variable.

6 With $s = (s_1, \ldots , s_N)$ representing a multi-dimensional scale parameter $s \in {\mathbb R}_+^N$, Eq. (7) should be interpreted as $\lim _{|s| \downarrow 0} L(\cdot ;\; s) = \lim _{|s| \downarrow 0} \mathcal{T}_s f = f$ with $|s| = \sqrt{s_1^2 + \cdots + s_N^2}$.

7 With $s_1 = (s_{1,1}, \dots , s_{1,N})$ and $s_2 = (s_{2,1}, \dots , s_{2,N})$ denoting two $N$-dimensional scale parameters, the inequality $s_2 \ge s_1$ should be interpreted as a requirement that the scale levels $s_1$ and $s_2$ have to be *ordered* in the sense that the increment $u = s_2 - s_1$ should correspond to a *positive direction* in parameter space that can be interpreted as increasing levels of scale. For example, for the affine spatial scale-space concept $L(x;\; \varSigma )$ to be considered later in Sect. 3, which for two-dimensional images $f$ can be parameterized by positive semi-definite $2 \times 2$ covariance matrices $\varSigma$, the requirement of an ordered and positive scale direction $u$ between the scale-space representations computed for two different covariance matrices $\varSigma _1$ and $\varSigma _2$ means that the difference between these covariance matrices $\varSigma _u = \varSigma _2 - \varSigma _1$ must be positive semi-definite. With the corresponding multi-dimensional scale parameters $s_1$ and $s_2$ expressed as vectors $s_1 = (\varSigma _{1,11}, \varSigma _{1,12}, \varSigma _{1,22})$ and $s_2 = (\varSigma _{2,11}, \varSigma _{2,12}, \varSigma _{2,22})$ where $\varSigma _{k,ij}$ denote the elements of $\varSigma _k$ for $k = 1$ and $2$, the condition for $u = (u_1, u_2, u_3) = s_2 - s_1$ to correspond to a positive direction in parameter space can therefore be expressed as $u_1 u_3 - u_2^2 \ge 0$ and $u_1 + u_3 \ge 0$.

8 This constant brightness assumption is guaranteed to hold for a Lambertian reflectance model extended with a spatially varying albedo, if the surface pattern is subject to illumination that is constant over time for corresponding surface points, see Sect. 2.3 for a more detailed model of receptive field responses under illumination variations. If the illumination intensity or the orientation of the surface normal in relation to the light source varies over time, however, the constant brightness assumption may be violated, or if the reflectance model comprises non-Lambertian, e.g., specular components. In such situations, a motion field computed from the optical flow obtained from the constant brightness assumption may therefore be different than the projected motion field of physical particles in the world. This situation can on the other hand be improved by instead applying a constancy assumption to spatial derivatives of the image intensity instead of the original zero-order image intensity. As explained in Sect. 2.3, such an assumption will in the specific case of a logarithmic brightness scale cancel the influence of local multiplicative illumination variations. By furthermore applying the constancy assumption to the output from several derivative operators simultaneously and additionally combining this assumption with an assumption of local coherence of the motion, e.g., in terms of a low parameter motion model over local regions in image space, one may additionally address the ambiguity of the aperture problem, provided that the local region of image space at which the low parameter image model is applied contains a sufficiently rich distribution of image structures of different orientations. Otherwise, the aperture problem states that under the assumption of constant brightness of corresponding physical points over time, only the motion component that is parallel to the local image gradient can be computed. The notion a Reichardt detector (Reichardt 1961; Reichardt and Schögl 1988) also addresses this issue by delay-coupled receptive fields in the retina. For the purpose of describing motion selective and motion-adapted properties of receptive fields, we shall, however, here for simplicity of presentation model temporal motions in terms of local Galilean transformations applied to image intensities, bearing in mind that this model can in a straightforward manner be transferred to the assumption of constancy of spatial derivative responses over time. Indeed, the spatio-temporal biological receptive fields that we shall describe in more detail in Sect. 6.3.2 do all support such a view by all comprising nonzero first, second, or third orders of spatial differentiation.

9 Note that the form of the vignetting effect may be different for lens systems composed of several lenses, and that lens systems are usually constructed to reduce the vignetting effect over some central part of the field of view. Notably, this natural vignetting effect will not be present with a spherical camera geometry, which is of high relevance with regard to biological vision.

10 To ensure sufficient differentiability properties such that an infinitesimal generator exists and the resulting multi-scale representation obtained by convolution with the semigroup of convolution kernels can be differentiated with respect to both space and scale such that the requirement of non-enhancement of local extrema can be applied, we do formally for an $N$-dimensional spatial domain require the semigroup $\mathcal{T}_s$ to be $C_1$-*continuous* such that $\lim _{h \downarrow 0} \left\| \frac{1}{h} \int _{s = 0}^{h} \mathcal{T}(s) f \, ds - f \right\| _{H^k({\mathbb R}^N)} = 0$ should hold for some $k > N/2$ and for all smooth functions $f \in L^1({\mathbb R}^N) \cap C^{\infty }({\mathbb R}^N)$ with $\Vert \cdot \Vert _{H^k({\mathbb R}^N)}$ denoting the $L^2$-based Sobolev norm $\Vert u \Vert _{H^k({\mathbb R}^N)} = \left( \int _{\omega \in {\mathbb R}^N} \left( 1 + |\omega |^2 \right) ^k \, |\hat{u}(\omega )|^2 \hbox {d}\omega \right) ^{1/2}$ and $\hat{u}$ denoting the Fourier transform of $u$ over ${\mathbb R}^N$; see (Lindeberg (2011), Sect. 3.2 and “Appendix A”) regarding details.

11 With “rotational invariance at the group level” meaning that although a set of receptive fields may not be rotationally symmetric as individuals, a collection or a group of such receptive fields may nevertheless make it possible to generate rotationally invariant responses, for example if all orientations are explicitly represented or if the receptive fields of different orientations can be related by linear combinations.

12 To ensure sufficient differentiability properties such that an infinitesimal generator exists and the resulting multi-scale representation obtained by convolution with the semigroup of convolution kernels can be differentiated with respect to both space–time and spatio-temporal scales such that the requirement of non-enhancement of local extrema can be applied, we do formally for an $N+1$-dimensional space–time require the semigroup $\mathcal{T}_s$ to be $C_1$-*continuous* in the sense that $\lim _{h \downarrow 0} \left\| \frac{1}{h} \int _{s = 0}^{h} \mathcal{T}(s) f \, ds - f \right\| _{H^k({\mathbb R}^N \times {\mathbb R})} = 0$ should hold for some $k > (N+1)/2$ and for all smooth functions $f \in L^1({\mathbb R}^N \times {\mathbb R}) \cap C^{\infty }({\mathbb R}^N \times {\mathbb R})$ with $\Vert \cdot \Vert _{H^k({\mathbb R}^2 \times {\mathbb R})}$ denoting the $L^2$-based Sobolev norm $\Vert u \Vert _{H^k({\mathbb R}^N \times {\mathbb R})} = \left( \int _{\omega \in {\mathbb R}^N \times {\mathbb R}} \left( 1 + |\omega |^2 \right) ^k \, |\hat{u}(\omega )|^2 \hbox {d}\omega \right) ^{1/2}$ and $\hat{u}$ denoting the Fourier transform of $u$ over ${\mathbb R}^N \times {\mathbb R}$; see (Lindeberg (2011), Sect. 3.2 and “Appendix A”) regarding details.

13 It can be shown that this definition is compatible with spatio-temporal scale invariance for scale selection based on local extrema over temporal scales of scale-normalized derivatives (manuscript in preparation). Specifically, the value $\kappa = 1/2$ can be motivated both from theoretical considerations and agreement with biological receptive fields.

14 By the use of locally adapted feedback, the resulting evolution equation does not obey the original linearity and shift invariance (homogeneity) requirements used for deriving the idealized affine Gaussian receptive field model, if the covariance matrices $\varSigma _0$ are determined from properties of the image data that are determined in a nonlinear way. For a fixed set of covariance matrices $\varSigma _0$ at any image point, the evolution equation will still be linear and will specifically obey non-enhancement of local extrema. In this respect, the resulting model could be regarded as a simplest form of nonlinear extension of the linear receptive field model.
